# Multi-omics analysis reveals the genetic basis for rapid CO_2_ utilization in the acetogenic bacterium *Sporomusa sphaeroides* KIAC

**DOI:** 10.1128/msystems.00451-25

**Published:** 2025-08-06

**Authors:** Jiyun Bae, Donghwi Lee, Chanho Park, Hyunwoo Jung, Minkyu Huh, Seulgi Kang, Ye Jin Gwak, Hyo Jung Lee, You-Jung Jung, Hyeokjun Yoon, Moonsuk Hur, Sangrak Jin, Suhyung Cho, Byung-Kwan Cho

**Affiliations:** 1 Department of Biological Sciences, Korea Advanced Institute of Science and Technology 34968, Daejeon, Republic of Korea; 2 Graduate School of Engineering Biology, Korea Advanced Institute of Science and Technology 34968, Daejeon, Republic of Korea; 3 Department of Biological Science, Kunsan National University 65450, Gunsan, Republic of Korea; 4 Biological and Genetic Resources Assessment Division, National Institute of Biological Resources 123590, Incheon, Republic of Korea; 5 Department of Biotechnology, Yeungnam University 35032, Gyeongbuk, Republic of Korea; 6 KI for the BioCentury, Korea Advanced Institute of Science and Technology 34968, Daejeon, Republic of Korea; Chinese Academy of Sciences, Shanghai, China

**Keywords:** acetogenic bacteria, CO_2 _fixation, hydrogenase, omics, regulatory systems, molecular mechanism

## Abstract

*Sporomusa sphaeroides* KIAC is a novel acetogen isolated from cattle feces that exhibits rapid CO_2_ utilization. To investigate the molecular basis of this phenotype, we performed a comprehensive multi-omics analysis, including Genome-seq, RNA-seq, dRNA-seq, and Term-seq, to map its transcriptome architecture. We identified 2,158 transcription start sites and 2,275 transcript 3′ ends, enabling high-resolution reconstruction of the transcriptional landscape and associated regulatory features. This analysis uncovered key *cis*-regulatory elements and an expanded regulatory role for the alternative sigma factor SigH in controlling acetogenesis-related genes. Notably, KIAC harbors nine functionally diverse hydrogenases—a greater diversity than observed in other acetogens—likely contributing to its rapid CO_2_ utilization. Heterologous expression of KIAC-derived hydrogenases in *Eubacterium limosum* led to doubled H_2_ and CO_2_ consumption rates, increased growth rates, and notably, the first reported butyrate production under energy-limited H_2_/CO_2_ conditions. These improvements stem from enhanced H_2_ oxidation, which supplies additional reducing equivalents for growth and biochemical production. Our findings provide critical insights into the genetic basis for rapid autotrophic growth in acetogens. The discovery of the expanded regulatory role of SigH and the energetic advantages of diverse hydrogenases offers new strategies for enhanced CO_2_ bioconversion of acetogens.

**IMPORTANCE:**

Acetogens offer a promising solution for sustainable CO_2_ bioconversion into multicarbon biochemicals through the Wood-Ljungdahl pathway, the most energy-efficient carbon fixation route known in nature. However, an incomplete understanding of their metabolism and regulatory systems has limited metabolic engineering efforts to achieve superior CO_2_ fixation efficiency. In this study, we investigated *Sporomusa sphaeroides* KIAC, a newly isolated acetogen with rapid CO_2_ utilization, to uncover the molecular mechanisms underlying its superior performance. By revealing an expanded regulatory role for an alternative sigma factor and a highly diverse set of hydrogenases, our findings provide a foundation for engineering acetogens with enhanced CO_2_ conversion efficiency under energy-limited conditions.

## INTRODUCTION

The Wood-Ljungdahl (WL) pathway represents the most energy-efficient CO_2_ fixation route in nature, requiring one ATP per acetyl-CoA produced (1). Acetogens utilizing this pathway are promising biocatalysts for sustainable CO_2_ conversion to multi-carbon biochemicals via acetyl-CoA (2, 3). While WL pathway overexpression and additional fixation routes have yielded modest improvements (less than twofold) (4–6), alternative strategies involve discovering novel acetogens with naturally superior CO_2_ utilization or screening relevant enzymes. A representative case of the latter approach is screening acetone biosynthetic enzymes from diverse ABE strains, which accelerated strain optimization and enhanced isopropanol production from waste gas in *Clostridium autoethanogenum* (7). These approaches not only circumvent strain engineering challenges but also provide valuable insights into efficient CO_2_ utilization mechanisms.

High-throughput sequencing technologies have advanced our understanding of regulatory systems in acetogens during autotrophic growth (8–11), revealing genetic regulation in *Acetobacterium bakii*, *Eubacterium limosum,* and *C. autoethanogenum* (10, 12–14). Despite sharing the WL pathway for carbon fixation, acetogens exhibit diverse metabolic strategies, phylogenetic variation, and bioenergetic differences (15, 16). Accordingly, transcriptional regulation of acetogenesis is also expected to vary across species. This diversity in regulatory systems highlights the need for further investigation. Integration of multi-omics data enables the construction of comprehensive transcriptome maps, defining functionally related transcription units and revealing key regulatory mechanisms (17–19). These insights provide data-driven strategies for developing more efficient CO_2_-fixing acetogens.

In this study, we report a comprehensive transcriptomic analysis of a newly isolated acetogen from cattle feces, *Sporomusa sphaeroides* KIAC, which exhibits rapid CO_2_ utilization. We generated high-resolution transcriptome maps revealing the molecular mechanisms underlying its CO_2_ utilization. Our analysis uncovered unique regulatory mechanisms, including an expanded role for the alternative sigma (σ) factor SigH in acetogenesis regulation. We also established a direct connection between multiple functionally diverse hydrogenases and efficient CO_2_ utilization, validated through heterologous expression in *E. limosum*. Notably, we observed butyrate production under energy-limited H_2_/CO_2_ conditions in batch cultures, a previously unreported phenotype. These findings provide the genetic basis for superior autotrophic growth in acetogens and engineering principles for enhancing CO_2_ utilization efficiency.

## RESULTS

### Genomic and phenotypic characterization of *Sporomusa sphaeroides* KIAC

**Fig 1:**
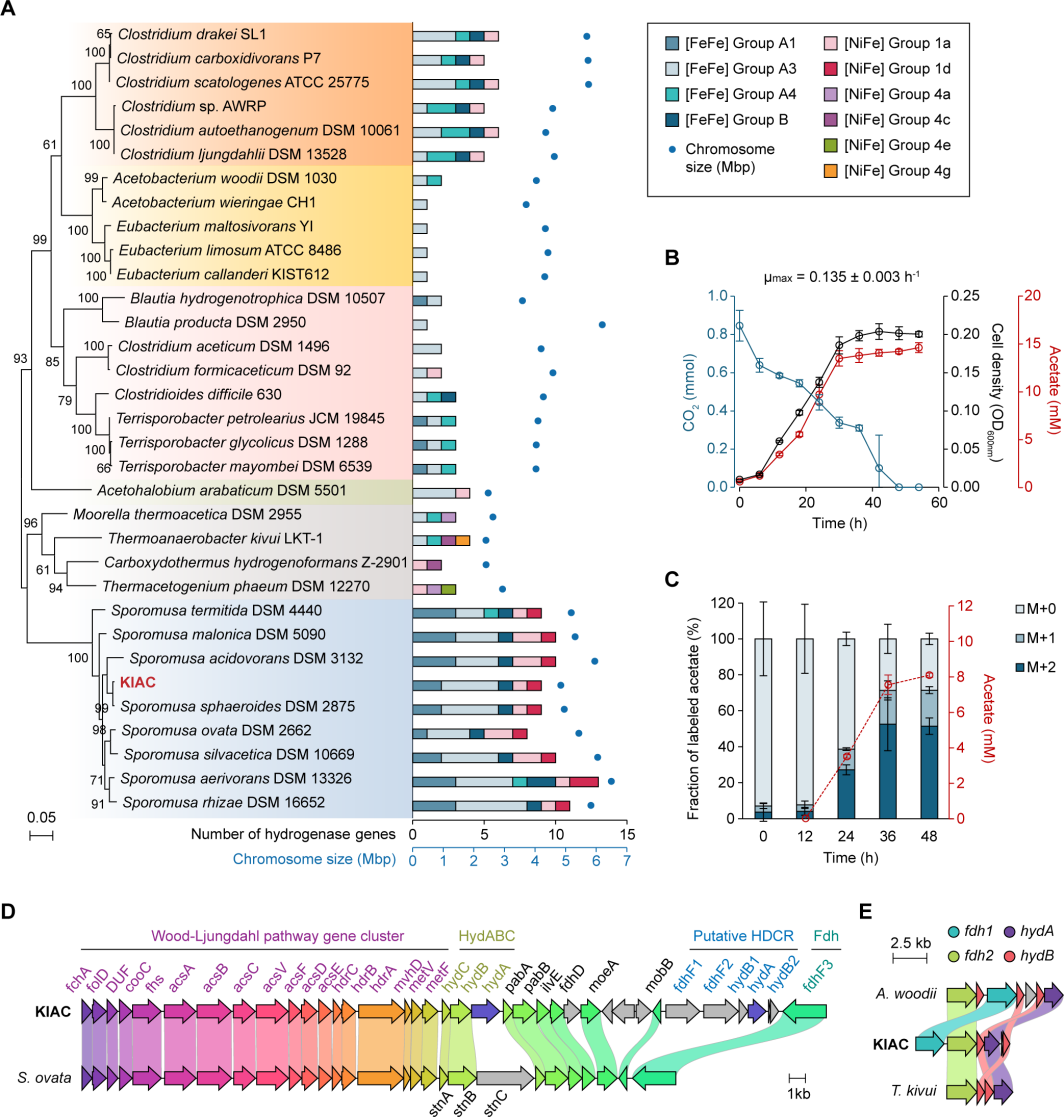
Comparative genomic analysis of the newly isolated acetogen *Sporomusa sphaeroides* KIAC. (**A**) 16S rRNA phylogenetic analysis of 32 acetogens and KIAC. The maximum-likelihood phylogenetic tree was constructed using the Tamura-Nei model in MEGA version 11 (20). Bootstrap values above 60 from 1,000 trials are shown at the nodes. The right graph compares the number of hydrogenase genes and chromosome sizes across acetogens. (**B**) Growth profile of KIAC cultivated under H_2_/CO_2_ conditions. Data are presented as mean ± SD from three biological replicates. The maximum growth rate (*µ*_max_) is shown above the panel. (**C**) Fraction of ^13^C-labeled acetate produced by KIAC during growth on H_2_/CO_2_. Bars represent the relative abundance of acetate mass isotopomers: M + 0 (unlabeled), M + 1 (singly labeled), and M + 2 (fully labeled with ^13^C from bicarbonate). Red circles and dashed lines indicate total acetate concentration over time. (**D**) Comparison of gene arrangement within the Wood-Ljungdahl pathway gene clusters and their downstream regions between *Sporomusa ovata* (SOV_RS07115-RS07250) and KIAC (KIAC18_000054-000091). Gene cluster comparison/visualization was performed using Clinker (21). Gene abbreviation information is listed in Data S1**.** (**E**) The putative H_2_-dependent CO_2_ reductase (HDCR) gene cluster of KIAC (KIAC18_000085-000090) compared to the HDCR gene clusters of *Acetobacterium woodii* and *Thermoanaerobacter kivui*.

We isolated a CO_2_-utilizing bacterium from Korean cattle feces and designated it as KIAC (see Method S1 for isolation details). To determine its genome, we used long-read PacBio and short-read Illumina sequencing, followed by *de novo* assembly. The genome consists of a single circular chromosome (4.84 Mbp and 47.4% GC content) encoding 4,496 genes, including 4,309 protein-coding genes, 33 rRNAs (5S, 16S, and 23S), 110 tRNAs, and 6 ncRNAs (Table S1). Phylogenetic analysis using 16S rRNA sequences placed KIAC close to *Sporomusa sphaeroides* DSM 2875 (Fig. 1A). Comparison with eight *Sporomusa* genomes revealed the highest similarity to *S. sphaeroides* with an average nucleotide identity (ANI) of 96.6%, exceeding the 95% species-level threshold (Fig. S1A). Accordingly, we designated the isolate as *S. sphaeroides* KIAC.

For phenotypic characterization, KIAC was cultivated in DSM 311 medium, commonly used for *Sporomusa*, containing 0.2% yeast extract as a complex component required to support its growth. Under H_2_/CO_2_ conditions, KIAC reached an OD_600_ of 0.204 ± 0.010 with a maximum growth rate of 0.135 ± 0.003 h^−1^, producing 14.62 ± 0.52 mM acetate as the sole product (Fig. 1B). CO_2_ was completely depleted within 48 h, consistent with the observed growth and metabolic activity. Compared to *S. sphaeroides* DSM 2875, KIAC exhibited a 1.6-fold higher growth rate and a 1.3-fold higher overall CO_2_ consumption rate (Fig. S1B and C), further distinguishing it from DSM 2875. The growth rate observed for KIAC is two- to threefold higher than those reported for several mesophilic acetogens, including *Clostridium drakei*, *E. limosum*, and *Sporomusa malonica* (0.04–0.07 h^−1^), and is comparable to that of *Acetobacterium woodii* (0.11 h^−1^) (Table S2 [5, 22–24]). Based on these comparisons, KIAC demonstrates relatively rapid growth under H_2_/CO_2_ conditions.

To verify CO_2_ fixation, particularly important given the presence of yeast extract as a potential alternative carbon source, we conducted ^13^C-isotope labeling analysis. KIAC was grown with ^13^C-labeled bicarbonate as the sole carbon source, replacing both unlabeled bicarbonate in the medium and gaseous CO_2_ in the headspace, followed by tracing its incorporation into acetate. The progressive accumulation of ^13^C-labeled acetate correlated with cellular growth (Fig. 1C), confirming CO_2_ fixation and its incorporation into acetate by KIAC.

### Comparative genomic analysis of *S. sphaeroides* KIAC

To gain insight into CO_2_ conversion in KIAC, we compared its WL pathway genes with those of *Sporomusa ovata*, the most well-characterized species in this genus, with a sequenced genome and relatively more research on acetogenic metabolism (25–29). In KIAC, WL pathway genes form a single consolidated gene cluster (Fig. 1D). The carbonyl branch genes (*cooC*, *acsA*, *acsB*, *acsC*, *acsV*, *acsF*, *acsD*, and *acsE*) are flanked by the methyl branch genes (*fchA*, *folD*, *fhs*, *hdrC*, *hdrB*, *hdrA*, *mvhD*, *metV*, and *metF*), with formate dehydrogenase (*fdhF1, fdh2,* and *fdhF3*) downstream. This organization resembles *Clostridium* species with minor variations, contrasting with the dispersed clusters in *E. limosum, A. woodii,* and *Moorella thermoacetica* (Fig. S2A). While acetogenesis-essential proteins in KIAC share over 82% identity with *S. ovata* homologs (Fig. S2B), distinct features appear downstream of its WL pathway gene cluster (Fig. 1D). Notably, KIAC lacks the typical *Sporomusa* type Nfn (Stn) transhydrogenase (25) and instead carries electron-bifurcating [FeFe]-hydrogenase genes (*hydABC*). It also contains three *fdh* genes: two selenocysteine-containing *fdhF1* and *fdhF3* (KIAC18_000085 and KIAC18_000091) and one non-selenocysteine *fdhF2* (KIAC18_000086). In contrast, *S. ovata* possesses two *fdh* genes, both homologous to *fdhF3* in KIAC, suggesting that *fdhF3* likely encodes a ferredoxin (Fd)-dependent Fdh (25). Unlike *fdhF3*, *fdhF1* and *fdhF2* lack N-terminal iron-sulfur clusters. A distinct genomic region (KIAC18_000085-000090) encompassing these *fdh* genes and [FeFe]-hydrogenase subunits (*hydA*, *hydB1,* and *hydB2*) is homologous to H_2_-dependent CO_2_ reductase (HDCR) systems in *A. woodii* and *Thermoanaerobacter kivui* (30, 31) (Fig. 1E), suggesting KIAC’s metabolic versatility in CO_2_ reduction and formate oxidation.

Analysis of 33 acetogen genomes revealed that hydrogenases are universally present, though their numbers and types vary (Fig. 1A). KIAC encodes nine hydrogenases: seven [FeFe]-hydrogenase (two Group A1, four Group A3, and one Group B) and two [NiFe]-hydrogenase (Groups 1a and 1d). This diversity is characteristic of *Sporomusa* species, with hydrogenase numbers correlating with chromosome size. Group A1 and A3 [FeFe]-hydrogenases dominate, facilitating H_2_ evolution and electron bifurcation from H_2_ to Fd and NAD(P), respectively (16). While [NiFe]-hydrogenases are less common in acetogens, Groups 1a and 1d are present in *Clostridium* and *Sporomusa* (Fig. 1A). In KIAC, [NiFe]-hydrogenase genes (KIAC18_000217-000219) are clustered with cytochrome *b*, resembling the energy conservation systems in *S. ovata*, where H_2_ oxidation is coupled to methylene-THF reduction, generating additional 0.5 ATP/methylene-THF via a cytochrome/quinone-dependent respiratory chain linked to the WL pathway (26). The diverse hydrogenase repertoire and auxiliary energy-conserving systems in KIAC likely enhance H_2_ oxidation efficiency, supply energy for CO_2_ reduction, and contribute to the low H_2_ threshold characteristic of *Sporomusa* species (15).

### Transcriptome architecture of *S. sphaeroides* KIAC

**Fig 2:**
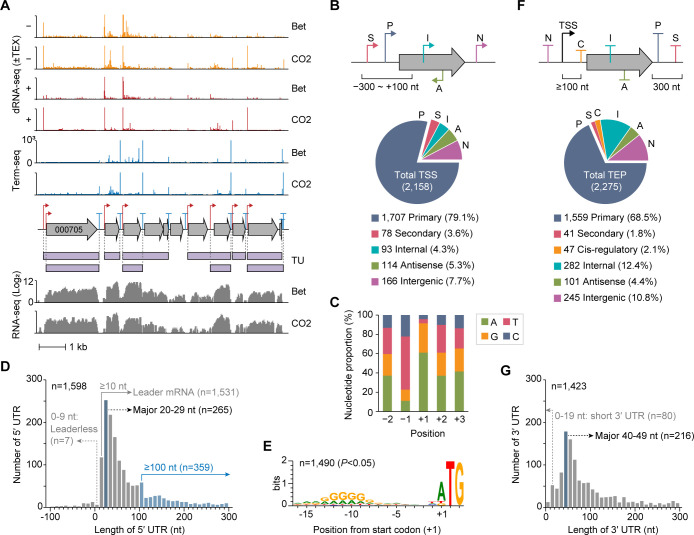
Transcriptome architecture of KIAC. (**A**) Example of dRNA-seq, Term-seq, and RNA-seq profiles mapped onto the KIAC genome. Each library was constructed from cultures grown on H_2_/CO_2_ (CO_2_) and betaine (Bet). For dRNA-seq, two libraries of TEX-treated (TEX+) and untreated RNA (TEX−) were constructed. (**B**) Classification of identified transcription start sites (TSSs) into five categories based on genomic positions relative to annotated genes: primary (P), secondary (S), internal (I), antisense (A), and intergenic (N) TSSs. (**C**) Nucleotide composition at TSS (+1) and surrounding −2 to +2 positions. (**D**) Distribution of 5′-UTR lengths associated with primary TSSs. (**E**) Conserved AG-rich ribosome binding site motif for 1,490 protein-coding transcripts with 5′-UTRs ≥ 10 nt. RBS motifs were detected from 20 nt upstream of start codons. (**F**) Classification of identified transcript 3′-end positions (TEPs) into six categories based on genomic positions relative to annotated genes, following the same method used for TSS classification, with the addition of a *cis*-regulatory (C) category. (**G**) Distribution of 3′-UTR lengths associated with primary TEPs. Detailed information on the identified TSSs and TEPs is provided in Data S2.

To characterize transcriptome architecture of KIAC, we employed dRNA-seq and Term-seq to detect transcription start and 3′-end positions under H_2_/CO_2_ and betaine conditions (32, 33) and integrated these with RNA-seq data to define transcription units (Fig. 2A; Table S3; Data S1).

dRNA-seq analysis identified highly reproducible 2,158 transcription start sites (TSSs; median Pearson’s *r* = 0.990), comprising 1,234 constitutive (57.2%), 643 CO_2_-specific (29.8%), and 281 betaine-specific (13.0%) sites (Table S4). Of these, 1,707 primary TSSs (79.1%) were located within 300 nt upstream to 100 nt downstream of gene 5′ ends, covering 36.5% of annotated genes (Fig. 2B; Data S2). An additional 78 secondary TSSs were identified in the same region with lower read counts, suggesting multiple TSSs for these genes. A total of 93 internal, 166 intergenic, and 114 antisense TSSs were also identified. Sequence analysis showed purine preference at the +1 position (61.1% A and 30.4% G) and pyrimidine preference at −1 (55.2% T and 22.1% C) (Fig. 2C), consistent with typical bacterial patterns. The median 5′-UTR length was 45 nt, with the most frequent length being 20–29 nt (Fig. 2D). Over 96% of transcripts were leader mRNAs containing 5′-UTRs longer than 10 nt, while only seven leaderless mRNAs were identified. A conserved AG-rich ribosome binding site (RBS) motif was detected in 97% of 5′-UTRs (Fig. 2E), consistent with other acetogens (10–12, 14).

Term-seq analysis identified 2,275 transcript 3′-end positions (TEPs; median Pearson’s *r* = 0.952), comprising 1,220 constitutive (53.6%), 532 CO_2_-specific (23.4%), and 523 betaine-specific (23.0%) positions (Table S4). Primary TEPs (68.5%) were within 300 nt of gene 3′ ends, covering 33.7% of total genes (Fig. 2F; Data S2). A total of 282 internal, 245 intergenic, 101 antisense, and 47 *cis*-regulatory TEPs were also identified. The median 3′-UTR length was 64 nt, peaking at 40–49 nt (Fig. 2G). Sequence analysis revealed typical intrinsic terminator features, such as U-rich regions and stem-loop structures (Text S1 and Fig. S3). While these features suggest potential Rho-independent termination, consistent with the absence of the Rho factor in KIAC, such intrinsic termination may also involve protein factors like NusA and NusG (34, 35).

Integrating all data sets, we inferred 2,417 putative transcription units, with overlapping units grouped into 850 transcription unit clusters (Text S2 and Fig. S4). Notably, WL pathway genes formed a single cluster (TUC-014, Data S2), and cobalamin riboswitches were the most abundant (Table S5), regulating genes for cobalamin biosynthesis and transport, supporting the essential role of cobalamin as a cofactor for WL pathway enzymes (36).

### Alternative sigma factors govern transcriptional regulation in KIAC

**Fig 3:**
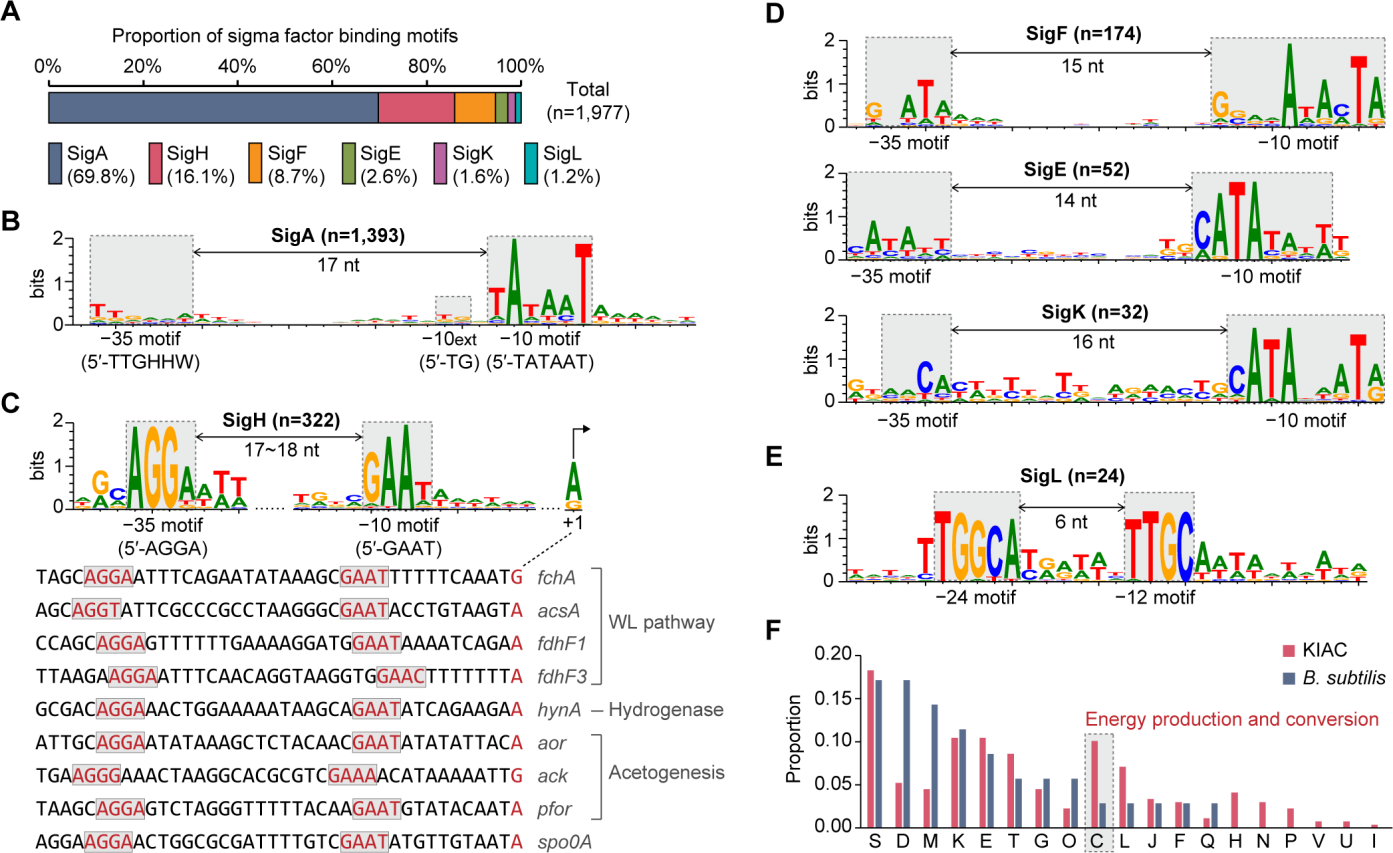
Sigma factor binding motifs detected by motif analysis of promoter sequences in KIAC. (**A**) Proportions of six sigma factor binding motifs detected in KIAC. Among the 2,158 identified TSSs, 80.6% (*n* = 1,740) contained at least one σ factor motif, with a total of 1,997 motifs identified. (**B**) The housekeeping SigA binding motif with −35 (TTGHHW) and −10 (TATAAT) elements separated by a 17-nt spacer. An extended −10 (−10ext, TG) element appeared 1 nt upstream of the −10 region. Binding motifs of (**C**) SigH and (**D**) SigF, SigE, and SigK. (**E**) The SigL binding motif with consensus −24 and −12 elements. For SigH binding motifs, 40 nt upstream of TSSs for acetogenesis-related genes and the well-known SigH-regulated *spo0A* are presented below the motif. (**F**) Comparison of COG functional category enrichment between SigH-regulated genes in *B. subtilis* and KIAC. COG functional categories are indicated by single-letter codes on the *x*-axis. S, function unknown; D, cell cycle control and cell division; M, cell wall/membrane/envelope biogenesis; K, transcription; E, amino acid transport and metabolism; T, signal transduction; G, carbohydrate transport and metabolism; O, post-translational modification, protein turnover, and chaperones; C, energy production and conversion; L, replication, recombination, and repair; J, translation; F, nucleotide transport and metabolism; Q, secondary metabolites biosynthesis; H, coenzyme metabolism; N, cell motility; P, inorganic ion transport and metabolism; V, defense mechanisms; U, intracellular trafficking; and I, lipid metabolism.

Analysis of 50 nt upstream sequences from TSSs using the MEME motif search algorithm (37) identified six conserved promoter motifs that closely resemble σ factor binding motifs in *Bacillus subtilis* (38). These motifs correspond to six σ factors (SigA, SigH, SigF, SigE, SigK, and SigL) (Fig. 3A through E; Data S2), reflecting the close homology between KIAC and *B. subtilis* σ factors (Table S6). The housekeeping SigA binding motif was predominant (69.8%), featuring conserved −10 (TATAAT), −35 (TTGHHW), and extended −10 (TG) motifs (Fig. 3B), comparable to *Escherichia coli* RpoD and *B. subtilis* SigA motifs (38, 39). SigH-binding motifs appeared in 322 TSSs (16.1%) (Fig. 3A), characterized by conserved −35 (AGGA) and −10 (GAAT) elements with 17–18 nt spacers (Fig. 3C), resembling *B. subtilis* and *Clostridium difficile* SigH regulon promoters (40, 41). SigF, SigE, and SigK binding motifs constituted 8.7%, 2.6%, and 1.6% of the total motifs, respectively (Fig. 3D), with spacer lengths matching *B. subtilis* patterns (38). These motifs consistently appeared in promoters of KIAC genes homologous to SigH, SigF, SigE, and SigK regulons in *B. subtilis* (42), including 32 genes involved in sporulation, sporulation response regulation, DNA replication, and cell division (Table S7).

Transcript levels of σ factors correlated with their target gene expression (Fig. S5A). SigA maintained consistent expression across conditions. SigH expression increased under H_2_/CO_2_ conditions, consistent with elevated target gene expression, supporting its role in autotrophic conditions. In contrast, SigF, SigE, and SigK were upregulated under betaine conditions, with SigK showing the most pronounced increase in both σ factor and target gene expression.

Unlike σ^70^ family members, SigL (RpoN) belongs to the σ^54^ family, recognizing −12/−24 elements and requiring bacterial enhancer-binding proteins (σ^54^-activators) for transcription initiation (43). We identified 24 promoters containing the conserved SigL motif (TGGCA-N_6_-TTGC) (Fig. 3E; Data S2). A 58 kb betaine metabolism gene cluster (KIAC18_004056-004100) contained multiple SigL-binding promoters and σ^54^-interacting transcriptional regulators (Fig. S5B). This cluster included genes for betaine transporters, betaine methyltransferase, trimethylamine methyltransferase, and glycine/sarcosine/betaine reductase, all specifically activated under betaine conditions (Data S2). A separate gene cluster containing *grdACDHI*, *trxAB*, *selAB*, *opuD3,* and a σ^54^-regulator showed SigA-dependent expression in both conditions. While previous studies identified σ^54^ roles in amino acid metabolism in *Clostridiales* (44), our finding is the first report of SigL-regulated betaine metabolism in KIAC.

### Potential role of SigH in regulating acetogenesis-related genes

The SigH regulon in KIAC includes 322 genes, establishing it as the second largest after SigA and significantly broader than the 48 gene SigH regulon in *B. subtilis* (40, 42). Clusters of Orthologous Groups (COG) analysis revealed both shared and distinct features between the two organisms. While both regulons are enriched in genes related to cell division (D), cell wall/membrane/envelope biogenesis (M), transcription (K), and amino acid metabolism (E), KIAC also shows notable enrichment in energy production (C) and six additional COG categories (H, N, P, V, U, and I) (Fig. 3F). This broader functional distribution suggests an expanded role of SigH, particularly in carbon and energy metabolism. Notably, several acetogenesis-related genes in KIAC contain SigH-binding motifs (Fig. 3C), including four WL pathway genes (*fchA*, *acsA*, *fdhF1*, and *fdhF3*), a [NiFe]-hydrogenase gene (*hynA*), and genes involved downstream of the WL pathway (*ack*, *aor*, and *pfor*). In other acetogens, *E. limosum*, *A. woodii*, and *A. bakii*, acetogenesis-related genes are primarily regulated by the housekeeping σ factor SigA (10–12), while *C. autoethanogenum* exhibits a novel SigA-driven promoter motif (14). Thus, SigH regulation of acetogenesis-related genes in KIAC represents a novel finding in acetogens.

To explore the broader relevance of SigH regulation, we searched for KIAC-like SigH-binding motifs within 200 nt upstream sequences of annotated genes across various acetogen genomes using FIMO (45). SigH motifs were detected in *Carboxydothermus hydrogenoformans*, *M. thermoacetica*, *Thermacetogenium phaeum*, *Acetohalobium arabaticum*, and all *Sporomusa* species, particularly upstream of WL pathway gene clusters, as observed in KIAC (Fig. S6 and S7). These acetogens share a highly conserved gene organization in the carbonyl branch of the WL pathway (*acsA*, *acsB*, *acsC*, *acsV*, *acsF*, *acsD*, and *acsE*). In contrast, *Clostridium*, *Terrisporobacter*, *Eubacterium*, *Acetobacterium*, and *T. kivui* showed fewer SigH motifs and lacked them in WL pathway genes, reflecting divergent promoter architectures (Fig. S6). These findings underscore the need for TSS determination in diverse acetogens to fully uncover the regulatory role of SigH.

### Transcriptomic changes under different growth conditions

**Fig 4:**
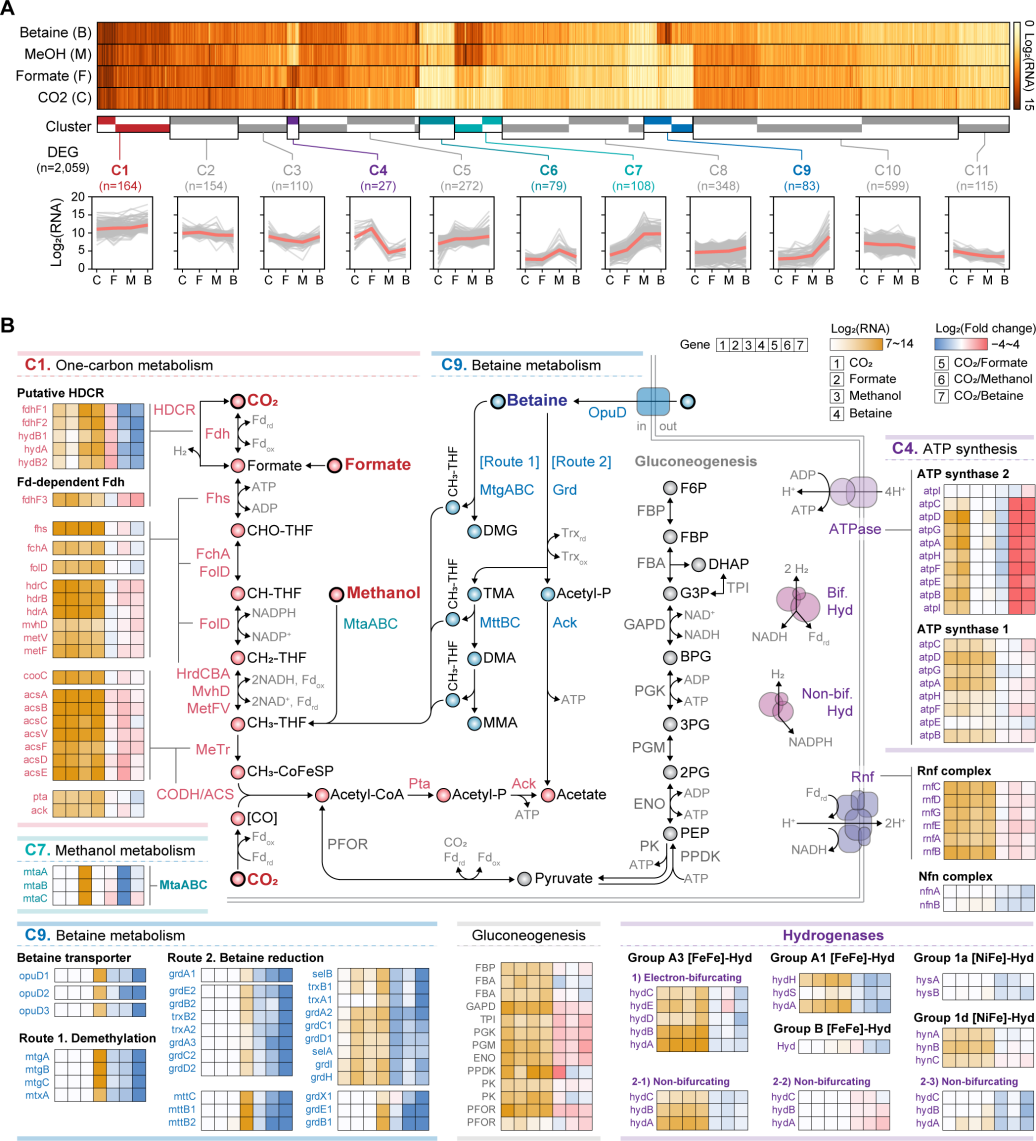
Transcriptomic and metabolic changes under four growth conditions, including H_2_/CO_2_, formate, methanol, and betaine. (**A**) Heatmap of differentially expressed genes (DEGs) showing significant changes in expression levels (log_2_ |fold change| > 1, adjusted *P*-value < 0.01) under two or more conditions. Data presented as log_2_-transformed normalized read counts. The resulting 2,059 DEGs were segregated into 11 groups of similar growth condition-specific expression patterns, as shown with clustered columns represented by colored bars. Gray and red lines indicate mean values of log_2_ (RNA) from three replicates and the median of each group, respectively. See also Data S1 for all transcript abundances. (**B**) Differential gene expression profiles in metabolic pathways of KIAC. (Top left) One-carbon metabolism, including the Wood-Ljungdahl pathway enriched in Cluster C1 and (center left) MtaABC involved in methanol metabolism, enriched in Cluster C7. (Top right) ATP synthase 2 enriched in Cluster C4. (Bottom left) Betaine metabolism enriched in Cluster C9. (Bottom center) Gluconeogenesis. (Bottom right) Hydrogenase genes, with four hydrogenases transcriptionally activated across all conditions. Fold changes of expression levels under H_2_/CO_2_ conditions are shown compared to formate, methanol, and betaine, respectively. Given the highly similar homologs of acetogenesis-related genes of KIAC with those of *S. ovata*, the metabolic pathways were constructed based on previously characterized one-carbon and betaine metabolisms of *S. ovata* (47, 48). For betaine metabolism, two possible pathways are shown with demethylation of betaine (Route 1) and reduction of betaine (Route 2). DMG, dimethylglycine; TMA, trimethylamine; DMA, dimethylamine; and MMA, monomethylamine. Information on gene abbreviations is available in Data S1.

RNA-seq analysis revealed acetogenesis-related transcriptome changes under four conditions (H_2_/CO_2_, formate, methanol, and betaine) (Fig. S8A through 8E and Table S3). Formate and methanol serve as direct C1 carbon and energy sources for the WL pathway (2), while betaine was used as a heterotrophic substrate (27, 46). Transcriptome analysis identified 2,059 differentially expressed genes (Data S1), grouped into 11 clusters, comprising condition-specific activation (C4, C6, C7, and C9) and multi-condition patterns (Fig. 4A).

Cluster C4, activated under H_2_/CO_2_ and formate, includes ATP synthase 2, which showed significantly higher expression than ATP synthase 1, suggesting the importance of chemiosmotic ATP generation during growth on oxidized C1 substrates (Fig. S9 and S4B, top right). Clusters C6 and C7 were methanol specific and contained corrinoid-associated proteins, including methanol:THF methyltransferase (MtaABC), encoded by the *mta* gene cluster in KIAC and organized similarly to that in *S. ovata* (Fig. S10A and S4B, center left). While *mtaR* and *mtaC1* were constitutively expressed, downstream genes (*mtaC2, mtaB, mtaA, mtaW,* and *mtaX*) showed methanol-specific upregulation (Fig. S10B), likely regulated by *mtaR* (49).

Cluster C9 encompasses betaine metabolism genes, most of which were specifically expressed under betaine conditions (Fig. 4B, bottom left). Betaine is utilized via two pathways: demethylation to dimethylglycine via methyltransferase (MtgABC) (47, 50, 51) or reduction to trimethylamine and acetyl-phosphate via glycine betaine reductase (Grd), followed by trimethylamine oxidation to CO_2_ (47, 51). In KIAC, these pathways are encoded within a 58 kb cluster, including genes for betaine transport (o*puD*), methyltransferase (*mtgABC*), trimethylamine methyltransferase (*mttBC*), glycine/sarcosine/betaine reductase (*grdABCDE* and *grdACDHI*), and selenocysteine incorporation (Fig. S10C and D).

### Hydrogenase expression and predicted functional roles in KIAC

Cluster C1 comprises genes with high transcript levels across four growth conditions (Fig. 4A). This cluster is enriched in carbon metabolism and fixation genes, particularly the WL pathway (Fig. S9; Fig. 4B, top left), indicating that carbons from CO_2_, formate, methanol, and betaine are metabolized through this pathway. Most WL pathway genes, along with genes encoding Rnf complex, ATP synthase 1, Pta, and Ack, were consistently expressed across all conditions, with a slight elevation under H_2_/CO_2_ and formate conditions. In contrast, *fdh* genes showed distinct patterns. The putative Fd-dependent Fdh showed approximately fivefold upregulation under H_2_/CO_2_ and formate, while the putative HDCR showed ~25-fold upregulation under methanol and betaine conditions. As in *A. woodii* (49), HDCR in KIAC may oxidize formate generated from methanol or betaine via the reverse WL pathway, producing H_2_ and enhancing reducing power. These patterns suggest that KIAC modulates formate oxidation routes based on the available substrate.

Four hydrogenases—one Group 1d [NiFe]-hydrogenase, one Group A1, and two Group A3 [FeFe]-hydrogenases—were constitutively expressed across conditions (Fig. 4B, bottom right). The [NiFe]-hydrogenase likely supports energy conservation via a respiratory chain (26). The [FeFe]-hydrogenases may serve dual roles: oxidizing H_2_ to supply reducing power for carbon fixation and reversibly generating H_2_ for redox balancing through NAD(P)H and Fd oxidation (49). Their expression, even without external H_2_, underscores the metabolic flexibility of KIAC.

While most WL pathway enzymes in KIAC likely rely on Fd or NADH, methylene-THF dehydrogenase is putatively NADPH-dependent based on its experimentally characterized homolog in *S. ovata* (25). Although KIAC encodes the Nfn complex (KIAC18_003409-003410), its low expression (Data S1) suggests alternative NADPH sources. Two Group A3 [FeFe]-hydrogenases may fulfill this role. HydABCDE (KIAC18_000110-000114) shares its genetic organization with the electron-bifurcating NAD-dependent hydrogenase of *A. woodii*, while HydABC (KIAC18_000072-000074) resembles the non-bifurcating hydrogenase of *Syntrophomonas wolfei* (Fig. S11A and S12). Structural predictions indicate NAD binding for HydABCDE and NADP binding for HydABC, consistent with their homologs (Fig. S11B and C). Together, they likely provide Fd, NADH, and NADPH to support WL pathway activity.

### Multiple hydrogenase functions enhance CO_2_ utilization during autotrophic growth

**Fig 5:**
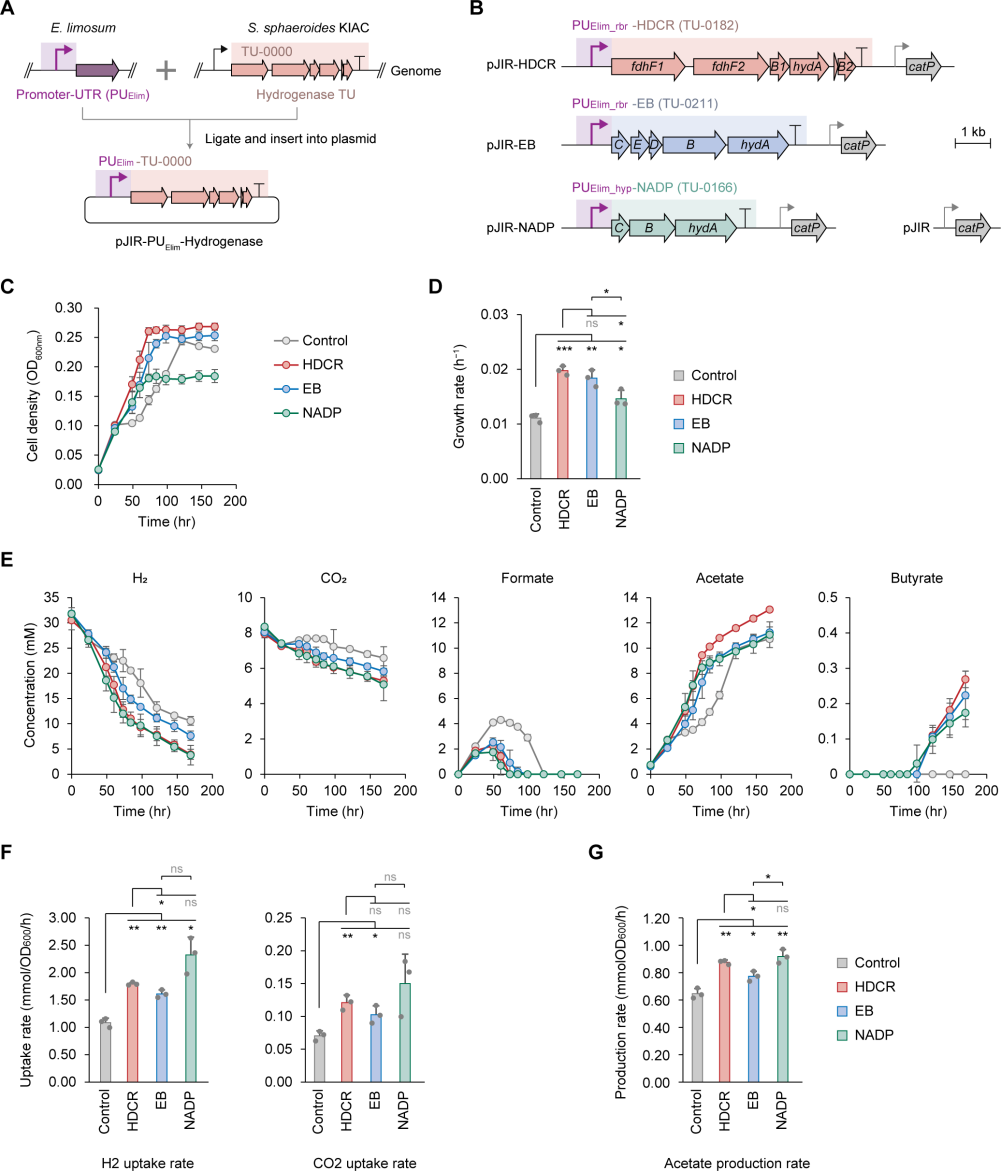
Phenotypical effects of introducing additional hydrogenases from *S. sphaeroides* KIAC into *E. limosum* on autotrophic growth. (**A**) Schematic of the construction strategy for plasmid-based expression of KIAC hydrogenases in *E. limosum*. To ensure proper transcription initiation, only the upstream promoter-UTR sequence of each KIAC hydrogenase transcription unit (TU) was replaced with a native constitutive promoter-UTR from *E. limosum* (PU_Elim_). Either one of two *E. limosum* promoter-UTR sequences was used: 200 bp upstream region of the rubrerythrin gene (*rbr*, B2M23_RS05660; PU_Elim_rbr_) for the HDCR and EB TUs, and that of the hypothetical protein gene (*hyp*, B2M23_RS12780; PU_Elim_hyp_) for the NADP TU. These promoters are known to drive high-level transcription and translation in *E. limosum* (13). The resulting constructs were ligated into a plasmid backbone to generate expression cassettes. (**B**) Genetic organization of hydrogenase expression plasmids introduced into *E. limosum*. Plasmids pJIR-HDCR, pJIR-EB, and pJIR-NADP harbor transcription units of putative HDCR, electron-bifurcating NAD-reducing hydrogenase, and non-bifurcating NADP-reducing hydrogenase, respectively, each under the control of PU_Elim_rbr_ or PU_Elim_hyp_. A control plasmid (pJIR) lacking hydrogenase genes was used as a negative control. (**C**) Cell growth and (**D**) growth rate of the engineered strains during growth under H_2_/CO_2_ conditions are shown. Growth rate (*µ*) represents *µ*_max_, calculated as the slope of the natural logarithm of OD_600_ values during the exponential growth phase. (**E**) Substrate (H_2_ and CO_2_) consumption and metabolite (formate, acetate, and butyrate) production profiles, (**F**) H_2_ and CO_2_ consumption rates, and (**G**) acetate production rate of each strain. Biomass-specific rates were calculated based on values obtained during the exponential phase and normalized to OD_600_ values. See Method S5 for detailed calculation method. Data are presented as mean ± SD from three biological replicates. Statistical significance was assessed for each strain in comparison to the control strain using Student’s *t* test (*P < 0.05; **P < 0.01; ***P < 0.001; and ns, not significant).

To investigate the roles of these hydrogenases in CO_2_ utilization, we heterologously expressed three transcriptionally active [FeFe]-hydrogenases from KIAC—the putative HDCR, electron-bifurcating hydrogenase (EB), and non-bifurcating NADP-reducing hydrogenase (NADP)—in the genetically accessible *E. limosum* harboring a single electron-bifurcating hydrogenase (Fig. 1A). Cytochrome-containing hydrogenases were excluded from this experiment due to the lack of cytochromes in *E. limosum*. Given the similarity in RBS and UTR features between KIAC and *E. limosum* (12, 13), only the upstream promoter-UTR of each hydrogenase transcription unit (TU-0182, TU-0211, and TU-0166) was replaced with a native *E. limosum* constitutive promoter-UTR (PU_Elim_) to ensure proper transcription initiation, while downstream UTRs from KIAC were retained (Fig. 5A; Table S8). The resulting plasmids (pJIR-HDCR, -EB, and -NADP) were introduced into *E. limosum*, generating hydrogenase-expressing strains (HDCR, EB, and NADP). A control strain was constructed using the plasmid backbone without hydrogenase genes (Fig. 5B).

Under H_2_/CO_2_ conditions, HDCR, EB, and NADP strains increased growth rates by 1.8-, 1.7-, and 1.3-fold, respectively, compared to the control (Fig. 5C and D). These strains also showed 1.5–2.1-fold higher H_2_ and CO_2_ uptake rates (Fig. 5E and F) and 1.2–1.4-fold increases in acetate production rates (Fig. 5E and G). Formate turnover, a key WL pathway step, occurred more rapidly in hydrogenase-expressing strains, indicating enhanced intracellular CO_2_ reduction, whereas the control strain showed slower turnover, likely due to limited reducing power from its single endogenous hydrogenase (Fig. 5E). Notably, only the engineered strains produced butyrate (0.2–0.3 mM) (Fig. 5E). This is the first observation in *E. limosum* under H_2_/CO_2_ batch conditions, previously seen only in bioreactors with high CO partial pressure (22, 52, 53). Although butyrate synthesis from H_2_/CO_2_ yields less energy (1 ATP/butyrate) than from CO (3.5 ATP/butyrate) (53), it is thermodynamically feasible with sufficient H_2_ supply (54), suggesting that KIAC-derived hydrogenases supplied excess reducing equivalents, enabling butyrate production as a redox-balancing mechanism.

qRT-PCR confirmed expression of the introduced hydrogenase genes only in the respective engineered strains (Fig. S13A), while endogenous hydrogenase levels remained constant (Fig. S13B), verifying that the observed phenotypes were conferred by KIAC-derived hydrogenases. Each hydrogenase appeared to impact *E. limosum* differently, depending on its redox cofactor specificity. In the EB strain, the introduced electron-bifurcating hydrogenase likely catalyzed the simultaneous reduction of NAD^+^ and ferredoxin during H_2_ oxidation. The increased supply of reduced ferredoxin would enhance ATP synthesis via the Rnf complex and ATP synthase, thereby improving energy conservation under autotrophic conditions (55).

In the NADP strain, the NADP-reducing hydrogenase likely elevated intracellular NADPH levels. This shift in the NADP^+^/NADPH ratio may have redirected reducing power toward redox-balancing or biosynthetic pathways. NADPH serves as an electron donor for 3-hydroxybutyryl-CoA dehydrogenase (Hbd), a key enzyme in butyrate formation in *E. limosum*, making butyrate formation thermodynamically favorable under NADPH excess (56). Despite lower biomass (OD_600_) than the control, the NADP strain produced butyrate (Fig. 5C and E), supporting the idea that increased NADPH redirected reducing equivalents toward metabolite synthesis rather than biomass formation. Unlike *E. limosum*, KIAC lacks a butyrate biosynthesis pathway and likely uses NADPH as a redox cofactor in WL pathway reactions (e.g., putatively NADP-dependent methylene-THF reductase). This suggests distinct roles of the NADP-reducing hydrogenase in the two strains, supporting redox balancing and product formation in *E. limosum* and enabling CO_2_ fixation in KIAC.

In the HDCR strain, the introduced HDCR likely facilitated direct CO_2_ reduction to formate via H_2_ oxidation, supplementing the native Fd-dependent Fdh in *E. limosum* (55). Although CO_2_ reduction using reduced ferredoxin is thermodynamically more favorable than using NADH or H_2_ (16), theoretical calculations have estimated that H_2_-dependent CO_2_ reduction yields more ATP (0.75 ATP/acetate) than Fd-dependent reduction (0.5 ATP/acetate) in *E. limosum* (53), indicating that HDCR may offer a more energetically efficient route for CO_2_ fixation. Additionally, diverting electrons away from Fd-dependent Fdh may also free up reduced ferredoxin to enhance ATP synthesis via the Rnf complex.

Collectively, these findings suggest that functionally diverse hydrogenases with distinct redox cofactor specificities enhance the redox flexibility and energy conservation of *E. limosum*, thereby improving H_2_/CO_2_ utilization. Notably, butyrate production was not solely due to increased H_2_ turnover but reflected altered redox cofactor availability, facilitated by the introduced hydrogenases. These results highlight the potential of employing multiple functionally diverse hydrogenases to improve CO_2_ and H_2_ utilization, supporting superior autotrophic growth capabilities observed in KIAC.

## DISCUSSION

We identified *S. sphaeroides* KIAC from cattle feces as a promising candidate, demonstrating rapid CO_2_ utilization with a growth rate of 0.135 h^−1^ and complete CO_2_ consumption within 48 h. Comprehensive multi-omics analysis, including Genome-seq, RNA-seq, dRNA-seq, and Term-seq, provided insights into its superior autotrophic growth. First, we identified a single consolidated WL pathway gene cluster, transcribed as a unified transcription unit. This contrasts with *E. limosum*, where the methyl branch, carbonyl branch, and *fdh* genes are regulated separately (12). More importantly, we uncovered the expanded role of alternative σ factor SigH in regulating acetogenesis-related genes, including the WL pathway. The potential involvement of SigH in phylogenetically related acetogens sharing conserved WL pathway gene organization suggests its broader importance in acetogenesis regulation. This functional divergence parallels the expanded roles of SigH in other bacteria, such as virulence and toxin gene expression in *Bacillus anthracis* (57) and *C. difficile* (41), and competence gene expression in non-sporulating bacteria *Listeria monocytogenes* (58) and *Lactobacillus sakei* (59). Given the phylogenetic diversity of acetogens harboring conserved WL pathways, this raises questions about diverse regulatory mechanisms governing acetogenesis.

We also established a direct link between hydrogenase diversity and rapid CO_2_ utilization in KIAC. Comparative genomic analysis revealed that *Sporomusa* species harbor a larger repertoire of hydrogenases than other acetogens. KIAC encodes nine functionally diverse hydrogenases, five of which were transcriptionally active, including a putative H_2_-dependent CO_2_ reductase, an electron-bifurcating NAD-reducing hydrogenase, a non-bifurcating NADP-reducing hydrogenase, and cytochrome-containing [FeFe]- and [NiFe]-hydrogenases. The identification of HDCR and non-bifurcating NADP-reducing hydrogenase is particularly notable, as HDCR has only been studied in *A. woodii* and *T. kivui* (30, 31), and the non-bifurcating NADP-reducing hydrogenase remains uncharacterized in acetogens.

To validate their functional roles, we heterologously expressed KIAC-derived hydrogenases in *E. limosum*, resulting in enhanced H_2_ and CO_2_ consumption, increased growth and acetate production, and, notably, the first observation of butyrate production under H_2_/CO_2_ conditions in batch cultures. This result suggests that KIAC hydrogenases increase H_2_ oxidation capacity, providing excess reducing equivalents for carbon fixation and redox balancing. These phenotypes likely reflect the exceptionally low H_2_ threshold (<20 Pa) characteristic of *Sporomusa* species (15), which enhances their suitability for gas fermentation and microbial electrosynthesis, where H_2_ availability is limited by its low solubility (1.6 mg/L at 293 K, 1 atm) (23, 28, 60).

Overall, this study expands our understanding of acetogenesis regulation and CO_2_ utilization mechanisms in *Sporomusa*, providing valuable insights for engineering acetogens with enhanced carbon fixation efficiency. Future research should focus on characterizing KIAC hydrogenases and determining whether their impact stems from superior catalytic activity or the synergistic effects of multiple functionally diverse hydrogenases. Additionally, experimental determination of TSSs in various acetogens would further illuminate the expanded regulatory roles of SigH and the potential contributions of other σ factors.

## MATERIALS AND METHODS

### Cultivation and physiological characterization of *Sporomusa sphaeroides* KIAC

*Sporomusa sphaeroides* KIAC has been deposited in the Korean Collection for Type Cultures (KCTC) with the accession number KCTC 19184P. KIAC was cultivated anaerobically at 37°C with 180 rpm agitation in 100 mL modified DSM 311 medium (pH 7.0). Casitone, betaine, sodium sulfide, and resazurin were omitted, while ammonium chloride was doubled to 1.0 g. The headspace was filled with H_2_/CO_2_ (80:20) at 200 kPa. For alternative growth conditions, either 43 mM betaine, 50 mM sodium formate, or 25 mM methanol was used, with an N_2_ headspace at 200 kPa. For analytical methods to measure substrates and metabolites, see Method S2.

### Genome and transcriptome sequencing

Genomic DNA was extracted with Qiagen Blood & Cell Culture DNA Mini Kit (Qiagen). The complete genome sequence of *S. sphaeroides* KIAC was obtained using PacBio Sequel II and Illumina NovaSeq 6000 (2 × 150 bp) platforms. *De novo* assembly with Flye (version 2.8.3) (61), circularization with Circlator (version 1.5.5) (62), and gene annotation using NCBI PGAP (63) were conducted. Total RNA was isolated from mid-exponential cultures using TRIzol (Thermo Scientific), and rRNA was depleted using RiboRid (64). RNA-seq libraries were prepared using the TruSeq Stranded mRNA Library Prep Kit (Illumina), while dRNA-seq and Term-seq libraries were constructed following published protocols with modifications (33, 64, 65). RNA-seq libraries were sequenced on Illumina MiSeq (2 × 75 bp), while dRNA-seq and Term-seq libraries were sequenced on Illumina NextSeq 1000 (1 × 100 bp). Sequencing data were processed and mapped to the KIAC genome using CLC Genomics Workbench 6.5.1 (Qiagen). Used primers are listed in Table S9. For detailed sequencing library preparation and data processing methods, see Method S3.

### Identification of hydrogenase genes across acetogens

Complete genome sequences of 32 acetogens were retrieved from NCBI (Table S10). Hydrogenases were identified by BLSATP against 3,277 hydrogenase protein sequences from HydDB (66) (*E*-value < 1e–20). To remove false positives, only hits annotated as “hydrogenase” were retained. Group C [FeFe] hydrogenases involved in H_2_ sensing and chemotaxis regulation were excluded. Consequently, 10 hydrogenase groups were detected across 33 acetogens.

### Identification of TSSs, TEPs, motifs, and transcription units

TSSs and TEPs were identified from uniquely mapped dRNA-seq and Term-seq reads, with slight modifications to previously described methods (18, 19, 65). Peaks were manually curated against RNA-seq profiles and classified by genomic positions. Results from two conditional libraries were merged within ±5 nt to generate total TSS and TEP lists (Data S2). Conserved motifs were identified using the MEME suite (37). SigH motifs were refined separately for −10 and −35 elements due to spacer length variations. Transcription units were determined as previously described by integrating TSS, TEP, and RNA-seq data (18, 19). One unit was defined as a connected region between TSS and TEP, supported by RNA-seq profiles to remove false positives. Overlapping units were grouped into clusters if they shared at least 1 nt. For details, see Method S4.

### Heterologous expression of hydrogenase genes from KIAC in *E. limosum*

Three selected [FeFe]-hydrogenases from KIAC were cloned into the pJIR750 plasmid using *E. coli* DH5α (Enzynomics). Gene fragments were amplified by PCR using Phusion High-Fidelity polymerase (Thermo) with primers (Table S9) and ligated into the plasmid using In-Fusion HD Cloning Kit (Takara Bio). Plasmids were transformed into *E. limosum* by electroporation, and resulting engineered strains were cultured at 37°C in DSMZ135 medium under H_2_/CO_2_ conditions, as previously described (13). Plasmid maintenance was ensured using 34 µg/mL chloramphenicol for *E. coli* and 15 µg/mL thiamphenicol for *E. limosum* (see Method S5 for experimental details).

## Data Availability

The complete genome sequence of *S. sphaeroides* KIAC has been deposited in GenBank under the accession number CP181154. Associated sequencing data (RNA-seq, dRNA-seq, and Term seq) were deposited to the SRA under the BioProject accession number PRJNA1214563.

## SUPPLEMENTAL MATERIAL

Data S1 msystems.00451-25-s0001.xlsx Transcriptome data.

Data S2 msystems.00451-25-s0002.xlsx List of total TSSs, TEPs, and TUs identified in KIAC.

Supplemental material msystems.00451-25-s0003.pdf Figures S1 to S13, Tables S1 to S10, Methods S1 to S5, and Texts S1 and S2.

ASM does not own the copyrights to Supplemental Material that may be linked to, or accessed through, an article. The authors have granted ASM a non-exclusive, world-wide license to publish the Supplemental Material files. Please contact the corresponding author directly for reuse.
