# Supplementary material for: Multi-omics analysis reveals the genetic basis for rapid CO2 utilization in the acetogenic bacterium Sporomusa sphaeroides KIAC
Source: mSystems. 2025 Aug 6;10(9):e00451-25. doi: 10.1128/msystems.00451-25 (PMC12456013; doi:10.1128/msystems.00451-25)
Supplement: Supplemental material — Figures S1 to S13, Tables S1 to S10, Methods S1 to S5, and Texts S1 and S2. [file msystems.00451-25-s0003.pdf]

Supplementary Information for

Multi-omics analysis reveals genetic basis for rapid CO_2 utilization in an acetogenic bacterium *Sporomusa sphaeroides* KIAC

Jiyun Bae[1], Donghwi Lee[1], Chanho Park[1], Hyunwoo Jung[1], Minkyu Huh[2], Seulgi Kang[1], Ye Jin Gwak[3], Hyo Jung Lee[3], You-Jung Jung[4], Hyeokjun Yoon[4], Moonsuk Hur[4], Sangrak Jin[1,5], Suhyung Cho[1,6], and Byung-Kwan Cho[1,2,6,*]

[1] Department of Biological Sciences, Korea Advanced Institute of Science and Technology, Daejeon 34141, Republic of Korea

[2] Graduate School of Engineering Biology, Korea Advanced Institute of Science and Technology, Daejeon 34141, Republic of Korea

[3] Department of Biological Science, Kunsan National University, Gunsan 54150, Republic of Korea

[4] Biological and Genetic Resources Assessment Division, National Institute of Biological Resources, Incheon 22689, Republic of Korea

[5] Department of Biotechnology, Yeungnam University, Gyeongbuk 38542, Republic of Korea

[6] KI for the BioCentury, Korea Advanced Institute of Science and Technology, Daejeon 34141, Republic of Korea

* Corresponding author: Byung-Kwan Cho (bcho@kaist.ac.kr)

CONTENTS

Supplementary Tables

Table S1. General features of the *Sporomusa sphaeroides* KIAC genome

Table S2. Comparison of growth rates of acetogens cultivated under H_2/CO_2 conditions

Table S3. Sequencing statistics for RNA-seq, dRNA-seq, and Term-seq

Table S4. Statistics of identified transcription start sites and transcript 3′-end positions

Table S5. Predicted ncRNAs from non-coding TUs using Rfam database

Table S6. Sigma factor homolog search results

Table S7. Homologous genes in KIAC with alternative sigma factor motifs similar to *B. subtilis*

Table S8. Transcription units of hydrogenase genes in KIAC

Table S9. List of oligonucleotides used in this study

Table S10. List of acetogenic bacteria with complete genome sequences

Supplementary Figures

Figure S1. Comparative genomic and phenotypic analysis of *S. sphaeroides* KIAC

Figure S2. Comparative analysis of acetogenesis-related genes in KIAC

Figure S3. Discovery of two groups of TEPs and their characteristics in KIAC

Figure S4. Determination of transcription units (TUs) and TU clusters in KIAC

Figure S5. Characteristics of alternative sigma factors and their binding motifs in KIAC

Figure S6. Occurrence of KIAC-like SigH binding motifs

Figure S7. Occurrence of SigH binding motifs across acetogens

Figure S8. RNA-seq analysis of KIAC under four different growth conditions

Figure S9. Enriched KEGG pathways for all differentially expressed genes

Figure S10. Alignment of genes involved in methanol and betaine metabolism

Figure S11. Comparison of [FeFe]-hydrogenases from KIAC

Figure S12. Multiple sequence alignment of HydB

Figure S13. Introduction of hydrogenases from *S. sphaeroides* KIAC into *E. limosum*

Supplementary Text

Text S1. Intrinsic termination prevails in Rho-deficient *S. sphaeroides* KIAC

Text S2. Transcription unit determination in KIAC

Supplementary Methods

Method S1. Enrichment and isolation of acetogenic bacteria

Method S2. Analytical methods

Method S3. Sequencing and data analysis

Method S4. Identification of TSSs, TEPs, motifs, and transcription units

Method S5. Heterologous expression of KIAC hydrogenases in *E. limosum*

Other Supplementary Data (Separate files)

Data S1. Transcriptome data

Data S2. List of total TSSs, TEPs, and TUs identified in KIAC

Table S1. General features of the *Sporomusa sphaeroides* KIAC genome.

Feature	Number (% of Total)
Scaffolds	1
Genome size (base pairs, bp)	4,844,680
GC content (%)	47.4
Total genes	4,496 (100.0%)
Coding sequences (CDSs)	4,347 (96.7%)
- Protein coding sequences	4,309 (95.8%)
- Pseudo genes (CDS without protein)	38 (0.8%)
RNA coding genes	149 (3.3%)
- rRNAs (5S, 16S, 23S)	33 (10, 12, 11) (0.7%)
- tRNAs	110 (2.4%)
- ncRNAs	6 (0.1%)
CRISPR repeat arrays	4

Table S2. Comparison of growth rates of acetogens cultivated under H_2/CO_2 conditions.

Acetogenic bacterium	Medium (complex component)	Culture medium volume	H_2/CO_2 composition, gas pressure	Growth rate (h^{-1})	Ref.*
Acetobacterium woodii DSMZ 1030	DSM 135 (2 g/L yeast extract)	20 ml in 120-ml serum bottle	64:16, 100 kPa	0.112	(1)
Acetobacterium woodii DSMZ 1030	DSM 135 (2 g/L yeast extract)	100 ml in 150-ml serum bottle	80:20, 200 kPa	0.050	(2)
Clostridium drakei SL1^T	DSM 135 (2 g/L yeast extract)	100 ml in 150-ml serum bottle	80:20, 200 kPa	0.044	(3)
Eubacterium limosum KIST612	Carbonate buffered basal medium	500 ml in serum bottle	80:20, 100 kPa	0.040	(4)
Sporomusa malonica DSM 5090	modified DSM 311 (0.3 g/L yeast extract)	100 ml in serum bottle	80:20, 1.7 atm	0.071	(5)
Sporomusa sphaeroides KIAC	modified DSM 311 (2 g/L yeast extract)	100 ml in 150-ml serum bottle	80:20, 200 kPa	0.135	This study

Ref. (1) Bertsch et al., 2015; (2) Shin et al., 2021; (3) Song et al., 2020; (4) Litty et al., 2021; (5) Aryal et al., 2017.

*All data were obtained from batch cultivations conducted in serum bottles.

Table S3. Sequencing statistics for RNA-seq, dRNA-seq, and Term-seq.

Sample name	Raw data		After trimming			Mapping (reference genome: *Sporomusa sphaeroides* KIAC)				
	Total reads	Avg. length	Trimmed reads	%	Avg. length	Mapped reads	%	Avg. length	Coverage (base)	rRNA %
RNA-seq (MiSeq, 2x75 bp)										
Betaine-1	6,539,022	75.5	6,531,208	99.9%	74.5	6,204,227	95.1%	74.9	95.9	0.16%
Betaine-2	9,499,910	75.5	9,480,684	99.8%	73.9	8,817,725	93.2%	74.6	135.7	0.04%
Betaine-3	8,356,974	75.5	8,347,224	99.9%	74.7	8,008,868	96.1%	75.0	123.9	0.03%
CO2-1	7,489,030	75.5	7,472,175	99.8%	73.9	6,912,941	92.7%	74.6	106.4	0.07%
CO2-2	6,617,134	75.5	6,603,828	99.8%	74.0	6,123,180	92.9%	74.6	94.3	0.08%
CO2-3	7,427,158	75.5	7,413,578	99.8%	74.1	6,898,592	93.2%	74.7	106.3	0.12%
For_1	6,960,404	75.5	6,950,805	99.9%	75.4	6,665,311	96.0%	75.5	103.9	0.25%
For_2	8,266,670	75.5	8,256,691	99.9%	75.5	8,004,707	97.1%	75.5	124.7	0.17%
For_3	8,516,858	75.5	8,506,330	99.9%	75.4	8,234,121	96.9%	75.5	128.3	0.16%
MeOH_1	7,938,342	75.5	7,928,442	99.9%	75.4	7,620,503	96.2%	75.4	118.6	0.35%
MeOH_2	7,184,964	75.5	7,176,555	99.9%	75.4	6,896,513	96.2%	75.4	107.4	0.45%
MeOH_3	8,898,654	75.5	8,887,858	99.9%	75.4	8,521,661	96.0%	75.5	132.7	0.31%
TSS-seq (NextSeq 1000, 1x100 bp)										
Betaine_TEX_1	18,332,054	96.9	17,551,808	95.7%	97.6	16,444,629	93.7%	99.1	336.4	0.09%
Betaine_TEX_2	16,245,591	98.0	15,828,344	97.4%	97.8	14,983,274	94.7%	99.4	307.3	0.08%
Betaine_TEX_3	14,864,292	98.4	14,555,056	97.9%	97.9	13,791,039	94.8%	99.5	283.3	0.06%
CO2_TEX_1	21,236,192	91.4	19,712,869	92.8%	93.6	18,498,341	93.8%	95.2	363.4	0.27%
CO2_TEX_2	31,068,785	87.1	27,043,637	87.0%	91.6	25,086,937	92.8%	94.0	486.5	0.35%
CO2_TEX_3	24,411,570	84.6	20,576,171	84.3%	89.2	18,457,242	89.7%	92.4	351.9	0.51%
Betaine_1	12,120,249	99.7	12,067,073	99.6%	99.4	11,719,471	97.1%	99.9	241.6	0.07%
Betaine_2	14,272,603	99.5	14,213,081	99.6%	99.1	13,829,394	97.3%	99.7	284.4	0.02%
Betaine_3	14,340,452	99.4	14,280,003	99.6%	98.8	13,873,053	97.2%	99.5	284.9	0.02%
CO2_1	15,009,198	97.5	14,632,404	97.5%	96.8	13,795,730	94.3%	98.6	280.8	0.09%
CO2_2	15,877,575	99.1	15,731,674	99.1%	98.5	15,113,510	96.1%	99.5	310.3	0.51%
CO2_3	28,798,328	92.7	27,279,646	94.7%	94.4	25,983,569	95.3%	95.3	510.9	0.14%
Term-seq (NextSeq 1000, 1x100 bp)										
Term_Betaine_1	21,508,905	87.4	18,735,452	87.1%	90.4	17,087,750	91.2%	93.5	329.6	1.00%
Term_Betaine_2	18,983,611	90.9	16,894,137	89.0%	93.4	15,919,897	94.2%	95.1	312.4	0.54%
Term_Betaine_3	19,726,674	86.6	16,895,059	85.7%	90.4	15,278,895	90.4%	93.3	294.4	0.75%
Term_CO2_1	24,391,806	91.8	21,882,851	89.7%	93.9	20,924,341	95.6%	95.0	410.3	0.22%
Term_CO2_2	18,442,456	88.9	15,931,717	86.4%	92.8	14,996,303	94.1%	94.5	292.6	0.43%
Term_CO2_3	21,574,480	89.3	18,982,569	88.0%	92.2	17,753,102	93.5%	94.0	344.6	0.44%

Table S4. Statistics of identified transcription start sites and transcript 3′-end positions.

**Transcription start sites (TSS)**	**Total TSS in each condition (n = 1,877; 1,515)**					**Shared TSS (n = 1,234)**			**Condition-specific TSS (n = 643; 281)**					**Total TSS (n = 2,158)**		
		**CO2**		**Betaine**		**CO2 & Betaine**				**CO2**		**Betaine**		**CO2 + Betaine**		
**Category**		**#**	**%**	**#**	**%**		**#**	**%**		**#**	**%**	**#**	**%**	**#**	**%**	
Primary (P)	P	1550	82.6	1206	79.6	P	1035	83.9	P	503	78.2	157	55.9	1707	79.1	a
Secondary (S)	S	46	2.5	49	3.2	S	5	0.4	S	27	4.2	32	11.4	78	3.6	b
Internal (I)	I	71	3.8	67	4.4	I	45	3.6	I	26	4.0	22	7.8	93	4.3	
Antisense (A)	A	63	3.4	87	5.7	A	36	2.9	A	27	4.2	51	18.1	114	5.3	
Intergenic (N)	N	147	7.8	106	7.0	N	87	7.1	N	60	9.3	19	6.8	166	7.7	
P in CO2 and S in Betaine	-	-	-	-	-	P/S	12	1.0	-	-	-	-	-	-	-	
S in CO2 and P in Betaine	-	-	-	-	-	S/P	14	1.1	-	-	-	-	-	-	-	
**Total**		**1877**	100	**1515**	100		**1234**	100		**643**	100	**281**	100	**2158**	100	

**Transcript 3′-end positions (TEP)**	**Total TEP in each condition (n = 1,752; 1,743)**					**Shared TEP (n = 1,220)**			**Condition-specific TEP (n = 532; 523)**					**Total TEP (n = 2,275)**		
		**CO2**		**Betaine**		**CO2 & Betaine**				**CO2**		**Betaine**		**CO2 + Betaine**		
**Category**		**#**	**%**	**#**	**%**		**#**	**%**		**#**	**%**	**#**	**%**	**#**	**%**	
Primary (P)	P	1253	71.5	1238	71.0	P	924	75.7	P	320	60.2	306	58.5	1559	68.5	a
Secondary (S)	S	23	1.3	34	2.0	S	7	0.6	S	12	2.3	18	3.4	41	1.8	b
Cis-regulatory (C)	C	43	2.5	23	1.3	C	19	1.6	C	17	3.2	4	0.8	47	2.1	c
Internal (I)	I	174	9.9	181	10.4	I	73	6.0	I	101	19.0	108	20.7	282	12.4	
Antisense (A)	A	64	3.7	78	4.5	A	41	3.4	A	23	4.3	37	7.1	101	4.4	
Intergenic (N)	N	195	11.1	189	10.8	N	136	11.1	N	59	11.1	50	9.6	245	10.8	
P in CO2 and S in Betaine	-	-	-	-	-	P/S	9	0.7	-	-	-	-	-	-	-	
S in CO2 and P in Betaine	-	-	-	-	-	S/P	4	0.3	-	-	-	-	-	-	-	
C in CO2 and P in Betaine	-	-	-	-	-	C/P	4	0.3	-	-	-	-	-	-	-	
C in CO2 and N in Betaine	-	-	-	-	-	C/N	3	0.2	-	-	-	-	-	-	-	
**Total**		**1752**	100	**1743**	100		**1220**	100		**532**	100	**523**	100	**2275**	100	

a, P(CO2,Bet)+P/S

b, S(CO2,Bet)+S/P

c, C(CO2,Bet)+C/P+C/N

Table S5. Predicted ncRNAs from non-coding TUs using Rfam database.

TU ID	Category	Rfam match	Rfam accession	E-value	ncTU category
TU-0003	Intergenic	-	-	-	Novel
TU-0010	Intergenic	-	-	-	Novel
TU-0011	Intergenic	-	-	-	Novel
TU-0308	Intergenic	-	-	-	Novel
TU-0309	Intergenic	-	-	-	Novel
TU-0324	Intergenic	-	-	-	Novel
TU-0339	Cis-regulatory	Cobalamin	RF00174	1.2E-27	Conserved
TU-0342	Intergenic	6S	RF00013	3.8E-12	Conserved
TU-0417	Cis-regulatory	-	-	-	Novel
TU-0481	Intergenic	Lysine	RF00168	1.7E-31	Conserved
TU-0482	Intergenic	Lysine	RF00168	1.7E-31	Conserved
TU-0483	Intergenic	Lysine	RF00168	1.7E-31	Conserved
TU-0511	Intergenic	-	-	-	Novel
TU-0512	Intergenic	-	-	-	Novel
TU-0568	Intergenic	-	-	-	Novel
TU-0615	Cis-regulatory	T-box	RF00230	3.4E-18	Conserved
TU-0617	Cis-regulatory	L20_leader	RF00558	9.2E-13	Conserved
TU-0619	Intergenic	T-box	RF00230	4.1E-11	Conserved
TU-0620	Intergenic	T-box	RF00230	1.7E-22	Conserved
TU-0633	Intergenic	-	-	-	Novel
TU-0635	Intergenic	T-box	RF00230	1.3E-31	Conserved
TU-0637	Intergenic	-	-	-	Novel
TU-0657	Intergenic	6S	RF00013	1.2E-18	Conserved
TU-0658	Intergenic	6S	RF00013	1.2E-18	Conserved
TU-0745	Cis-regulatory	FMN	RF00050	5.1E-27	Conserved
TU-0760	Cis-regulatory	SAM	RF00162	3.4E-20	Conserved
TU-0761	Cis-regulatory	SAM	RF00162	3E-18	Conserved
TU-0771	Intergenic	-	-	-	Novel
TU-0775	Cis-regulatory	T-box	RF00230	4.3E-20	Conserved
TU-0796	Intergenic	-	-	-	Novel
TU-0797	Intergenic	-	-	-	Novel
TU-0798	Intergenic	-	-	-	Novel
TU-0805	Intergenic	-	-	-	Novel
TU-0814	Intergenic	T-box	RF00230	1.4E-26	Conserved
TU-0815	Intergenic	T-box	RF00230	7.5E-27	Conserved
TU-0816	Intergenic	T-box	RF00230	7.5E-27	Conserved
TU-0827	Cis-regulatory	T-box	RF00230	1.4E-32	Conserved
TU-0843	Intergenic	-	-	-	Novel

TU-0866	Cis-regulatory	-	-	-	Novel
TU-0867	Intergenic	-	-	-	Novel
TU-0882	Intergenic	-	-	-	Novel
TU-0883	Intergenic	-	-	-	Novel
TU-0884	Intergenic	-	-	-	Novel
TU-0888	Cis-regulatory	-	-	-	Novel
TU-0902	Cis-regulatory	c-di-GMP-I	RF01051	2.9E-16	Conserved
TU-0904	Intergenic	-	-	-	Novel
TU-0915	Cis-regulatory	-	-	-	Novel
TU-0918	Intergenic	-	-	-	Novel
TU-0922	Intergenic	-	-	-	Novel
TU-0923	Intergenic	-	-	-	Novel
TU-0926	Cis-regulatory	-	-	-	Novel
TU-0970	Cis-regulatory	SAM	RF00162	7E-20	Conserved
TU-0976	Cis-regulatory	-	-	-	Novel
TU-0977	Cis-regulatory	-	-	-	Novel
TU-0979	Intergenic	T-box	RF00230	1.6E-27	Conserved
TU-0992	Intergenic	BsrG	RF01412	4.7E-15	Conserved
TU-0993	Intergenic	BsrG	RF01412	4.7E-15	Conserved
TU-0994	Intergenic	BsrG	RF01412	4.7E-15	Conserved
TU-0996	Intergenic	BsrG	RF01412	3.7E-09	Conserved
TU-0998	Intergenic	Cobalamin	RF00174	1.4E-30	Conserved
TU-0999	Intergenic	Cobalamin	RF00174	1.9E-32	Conserved
TU-1000	Intergenic	Cobalamin	RF00174	1.9E-32	Conserved
TU-1001	Intergenic	Cobalamin	RF00174	1.9E-32	Conserved
TU-1002	Intergenic	Cobalamin	RF00174	1.6E-30	Conserved
TU-1003	Intergenic	Cobalamin	RF00174	1.9E-32	Conserved
TU-1004	Intergenic	Cobalamin	RF00174	1.9E-32	Conserved
TU-1005	Intergenic	Cobalamin	RF00174	1.9E-32	Conserved
TU-1006	Intergenic	Cobalamin	RF00174	1.6E-30	Conserved
TU-1007	Intergenic	Cobalamin	RF00174	1.2E-30	Conserved
TU-1008	Intergenic	Cobalamin	RF00174	1.2E-30	Conserved
TU-1009	Intergenic	Cobalamin	RF00174	1.2E-30	Conserved
TU-1010	Intergenic	Cobalamin	RF00174	4E-29	Conserved
TU-1028	Intergenic	-	-	-	Novel
TU-1029	Intergenic	-	-	-	Novel
TU-1098	Cis-regulatory	-	-	-	Novel
TU-1112	Intergenic	-	-	-	Novel
TU-1117	Cis-regulatory	-	-	-	Novel
TU-1132	Cis-regulatory	raiA	RF03072	6.2E-53	Conserved
TU-1136	Intergenic	-	-	-	Novel

TU-1158	Intergenic	-	-	-	Novel
TU-1159	Intergenic	-	-	-	Novel
TU-1162	Intergenic	-	-	-	Novel
TU-1163	Intergenic	-	-	-	Novel
TU-1189	Intergenic	-	-	-	Novel
TU-1208	Intergenic	-	-	-	Novel
TU-1303	Intergenic	-	-	-	Novel
TU-1304	Intergenic	-	-	-	Novel
TU-1323	Intergenic	group-II-D1D4-6	RF02005	1.5E-39	Conserved
TU-1346	Intergenic	-	-	-	Novel
TU-1347	Intergenic	-	-	-	Novel
TU-1371	Intergenic	-	-	-	Novel
TU-1376	Cis-regulatory	T-box	RF00230	8.2E-27	Conserved
TU-1379	Intergenic	-	-	-	Novel
TU-1390	Intergenic	BsrG	RF01412	1.7E-14	Conserved
TU-1391	Intergenic	BsrG	RF01412	1.7E-14	Conserved
TU-1392	Intergenic	BsrG	RF01412	1.7E-14	Conserved
TU-1395	Intergenic	-	-	-	Novel
TU-1396	Intergenic	-	-	-	Novel
TU-1412	Cis-regulatory	-	-	-	Novel
TU-1414	Cis-regulatory	-	-	-	Novel
TU-1422	Cis-regulatory	-	-	-	Novel
TU-1424	Cis-regulatory	-	-	-	Novel
TU-1446	Intergenic	Cobalamin	RF00174	1E-31	Conserved
TU-1459	Intergenic	T-box	RF00230	6.5E-27	Conserved
TU-1462	Intergenic	-	-	-	Novel
TU-1465	Intergenic	-	-	-	Novel
TU-1469	Intergenic	-	-	-	Novel
TU-1471	Intergenic	-	-	-	Novel
TU-1487	Intergenic	T-box	RF00230	3.2E-29	Conserved
TU-1488	Intergenic	T-box	RF00230	1.3E-24	Conserved
TU-1489	Intergenic	Cobalamin	RF00174	1.6E-19	Conserved
TU-1490	Intergenic	Cobalamin	RF00174	1.7E-18	Conserved
TU-1536	Intergenic	Cobalamin	RF00174	2.3E-30	Conserved
TU-1537	Intergenic	Cobalamin	RF00174	2.3E-30	Conserved
TU-1538	Intergenic	-	-	-	Novel
TU-1539	Intergenic	-	-	-	Novel
TU-1540	Intergenic	-	-	-	Novel
TU-1543	Intergenic	-	-	-	Novel
TU-1544	Intergenic	-	-	-	Novel
TU-1545	Intergenic	-	-	-	Novel

TU-1548	Intergenic	-	-	-	Novel
TU-1555	Cis-regulatory	-	-	-	Novel
TU-1581	Cis-regulatory	-	-	-	Novel
TU-1582	Cis-regulatory	-	-	-	Novel
TU-1605	Cis-regulatory	Cobalamin	RF00174	2.1E-29	Conserved
TU-1606	Cis-regulatory	Cobalamin	RF00174	2.1E-29	Conserved
TU-1626	Intergenic	T-box	RF00230	1.3E-23	Conserved
TU-1642	Intergenic	-	-	-	Novel
TU-1643	Intergenic	-	-	-	Novel
TU-1644	Intergenic	-	-	-	Novel
TU-1645	Intergenic	-	-	-	Novel
TU-1646	Intergenic	-	-	-	Novel
TU-1670	Intergenic	-	-	-	Novel
TU-1671	Intergenic	-	-	-	Novel
TU-1692	Intergenic	-	-	-	Novel
TU-1695	Cis-regulatory	-	-	-	Novel
TU-1713	Cis-regulatory	-	-	-	Novel
TU-1714	Cis-regulatory	-	-	-	Novel
TU-1715	Intergenic	-	-	-	Novel
TU-1717	Cis-regulatory	TPP	RF00059	3.3E-15	Conserved
TU-1766	Cis-regulatory	-	-	-	Novel
TU-1767	Cis-regulatory	-	-	-	Novel
TU-1774	Intergenic	-	-	-	Novel
TU-1775	Intergenic	-	-	-	Novel
TU-1798	Cis-regulatory	-	-	-	Novel
TU-1799	Cis-regulatory	-	-	-	Novel
TU-1800	Cis-regulatory	SAM	RF00162	1.4E-23	Conserved
TU-1804	Intergenic	T-box	RF00230	3.8E-20	Conserved
TU-1807	Intergenic	T-box	RF00230	4.6E-22	Conserved
TU-1837	Intergenic	-	-	-	Novel
TU-1881	Cis-regulatory	-	-	-	Novel
TU-1882	Cis-regulatory	-	-	-	Novel
TU-1884	Intergenic	-	-	-	Novel
TU-1885	Intergenic	-	-	-	Novel
TU-1889	Cis-regulatory	-	-	-	Novel
TU-1904	Cis-regulatory	-	-	-	Novel
TU-1925	Intergenic	-	-	-	Novel
TU-1933	Cis-regulatory	-	-	-	Novel
TU-1934	Intergenic	Cobalamin	RF00174	4.7E-27	Conserved
TU-1935	Intergenic	Cobalamin	RF00174	4.7E-27	Conserved
TU-1937	Cis-regulatory	-	-	-	Novel

TU-1979	Cis-regulatory	-	-	-	Novel
TU-1984	Intergenic	-	-	-	Novel
TU-1999	Intergenic	T-box	RF00230	4.3E-23	Conserved
TU-2027	Cis-regulatory	-	-	-	Novel
TU-2083	Intergenic	-	-	-	Novel
TU-2084	Intergenic	-	-	-	Novel
TU-2085	Intergenic	-	-	-	Novel
TU-2097	Cis-regulatory	-	-	-	Novel
TU-2115	Intergenic	Cobalamin	RF00174	2.3E-31	Conserved
TU-2116	Intergenic	Cobalamin	RF00174	2.3E-31	Conserved
TU-2117	Intergenic	Cobalamin	RF00174	7.6E-30	Conserved
TU-2118	Intergenic	Cobalamin	RF00174	7.6E-30	Conserved
TU-2120	Intergenic	Cobalamin	RF00174	5.8E-31	Conserved
TU-2121	Intergenic	Cobalamin	RF00174	5.5E-31	Conserved
TU-2122	Intergenic	Cobalamin	RF00174	5.5E-31	Conserved
TU-2123	Intergenic	Cobalamin	RF00174	5.5E-31	Conserved
TU-2124	Intergenic	Cobalamin	RF00174	3.4E-33	Conserved
TU-2125	Intergenic	Cobalamin	RF00174	3.4E-33	Conserved
TU-2126	Cis-regulatory	Cobalamin	RF00174	4.5E-12	Conserved
TU-2129	Intergenic	Cobalamin	RF00174	1.1E-31	Conserved
TU-2130	Intergenic	Cobalamin	RF00174	1.1E-31	Conserved
TU-2131	Intergenic	Cobalamin	RF00174	1.5E-31	Conserved
TU-2132	Intergenic	Cobalamin	RF00174	3.5E-31	Conserved
TU-2133	Intergenic	Cobalamin	RF00174	3.5E-31	Conserved
TU-2136	Cis-regulatory	-	-	-	Novel
TU-2137	Cis-regulatory	-	-	-	Novel
TU-2138	Cis-regulatory	-	-	-	Novel
TU-2248	Cis-regulatory	SAM	RF00162	9.6E-23	Conserved
TU-2262	Intergenic	Cobalamin	RF00174	1.9E-25	Conserved
TU-2270	Intergenic	-	-	-	Novel
TU-2271	Intergenic	-	-	-	Novel
TU-2276	Intergenic	-	-	-	Novel
TU-2298	Intergenic	-	-	-	Novel
TU-2311	Intergenic	-	-	-	Novel
TU-2312	Intergenic	-	-	-	Novel
TU-2313	Intergenic	-	-	-	Novel
TU-2314	Intergenic	-	-	-	Novel
TU-2316	Intergenic	-	-	-	Novel
TU-2350	Intergenic	-	-	-	Novel
TU-2353	Cis-regulatory	-	-	-	Novel
TU-2361	Intergenic	-	-	-	Novel

TU-2362	Intergenic	-	-	-	Novel
TU-2363	Intergenic	-	-	-	Novel
TU-2364	Intergenic	-	-	-	Novel
TU-2365	Intergenic	-	-	-	Novel
TU-2373	Intergenic	-	-	-	Novel
TU-2380	Cis-regulatory	-	-	-	Novel
TU-2402	Intergenic	T-box	RF00230	6.2E-25	Conserved
TU-2412	Intergenic	-	-	-	Novel

Table S6. Sigma factor homolog search results.

Locus tag	Annotation	Sigma factor	BLASTP results		
			Homologs (top 3 hits)	% identity	Evalue
KIAC18_000231	sigma-70 family RNA polymerase sigma factor	SigK	SigK of *B. subtilis*	47.1	1.27E-52
			SigE of *B. subtilis*	59.7	9.75E-52
			SigK of *C. difficile*	59.7	2.26E-45
KIAC18_000411	RNA polymerase sporulation sigma factor SigE	SigE	SigE of *B. subtilis*	70.8	1.12E-116
			SigE of *C. difficile*	69.1	1.75E-111
			SigK of *C. difficile*	48.3	8.12E-60
KIAC18_000412	RNA polymerase sporulation sigma factor SigG	SigG	SigG of *B. subtilis*	74.4	1.98E-142
			SigG of *C. difficile*	67.3	9.13E-134
			SigF of *B. subtilis*	47.9	2.06E-80
KIAC18_000509	FliA/WhiG family RNA polymerase sigma factor	SigD	SigD of *B. subtilis*	38.4	3.3E-54
			FliA of *E. coli*	39.5	4.56E-54
			FliA of *C. difficile*	34.5	1.64E-40
KIAC18_000593	SigB/SigF/SigG family RNA polymerase sigma factor	SigF	SigF of *B. subtilis*	50.4	3.29E-81
			SigG of *C. difficile*	47.5	2.84E-78
			SigG of *B. subtilis*	48.8	3.27E-76
KIAC18_000695	RNA polymerase sigma factor RpoD	SigA	SigA of *B. subtilis*	70.6	0
			SigA of *C. difficile*	68.5	0
			RpoS of *E. coli*	46.3	1.52E-80
KIAC18_001702	RNA polymerase factor sigma-54	SigL	SigL of *C. difficile*	35.6	5.32E-90
			RpoN of *E. coli*	34.0	1.64E-84
			SigL of *B. subtilis*	32.9	6.87E-70
KIAC18_003587	RNA polymerase sporulation sigma factor SigH	SigH	SigH of *B. subtilis*	75.1	5.57E-108
			SigH of *C. difficile*	70.7	3E-96

*Sigma factors from KIAC were compared with well-characterized sigma factors from *B. subtilis, C. difficile*, and *E. coli*.

Table S7. Homologous genes in KIAC with alternative sigma factor motifs similar to *B. subtilis*

KIAC genes*	*B. subtilis* homologs identified by blastp search (E-value < 1e-10)				Regulon in *B. subtilis*					Detected motifs in KIAC					Match
Locus tag	Gene	Locus tag	PID %	E-value	Sig A	Sig H	Sig F	Sig E	Sig K	Sig A	Sig H	Sig F	Sig E	Sig K	
KIAC18_000156	*minC*	BSU_28000	33.8	1.15E-26	O	O				O	O				SigA, SigH
KIAC18_000408	*ftsA*	BSU_15280	26.7	9.01E-32	O	O					O				SigH
KIAC18_000410	*spoIIGA*	BSU_15310	24.0	7.06E-20	O		O			O		O			SigA, SigF
KIAC18_000412	*sigG*	BSU_15330	74.4	1.74E-142	O		O			O		O			SigA, SigF
KIAC18_000414	*ylmC*	BSU_15360	54.1	6.56E-23				O		O			O		SigE
KIAC18_000424	*spoVV*	BSU_15030	45.2	1.68E-101				O					O		SigE
KIAC18_000441	*spoVS*	BSU_16980	75.6	2.54E-45		O					O				SigH
KIAC18_000566	*spoVS*	BSU_16980	93.0	3.50E-53		O					O				SigH
KIAC18_000579	*spo0A*	BSU_24220	57.5	5.00E-113	O	O					O				SigH
KIAC18_000591	*spoIIAA*	BSU_23470	48.0	3.31E-32		O	O				O				SigH
KIAC18_000607	*gerW*	BSU_29500	47.4	1.52E-36			O			O		O			SigF
KIAC18_000656	*gpr*	BSU_25540	41.8	7.13E-92			O			O		O			SigF
KIAC18_000657	*spoIIP*	BSU_25530	28.0	1.20E-19			O	O				O	O		SigF, SigE
KIAC18_000676	*yqfC*	BSU_25360	39.1	9.08E-20				O					O		SigE
KIAC18_000694	*dnaG*	BSU_25210	35.6	5.37E-109	O	O				O	O				SigA, SigH
KIAC18_000942	*mblK*	BSU_10750	38.7	1.75E-47		O				O	O				SigH
KIAC18_001748	*hpf*	BSU_35310	47.4	4.52E-56		O				O	O				SigH
KIAC18_001995	*spoIIID*	BSU_36420	58.5	4.51E-29				O					O		SigF
KIAC18_001997	*spoIID*	BSU_36750	36.7	6.24E-66				O		O			O		SigF
KIAC18_002000	*murAA*	BSU_36760	61.2	0	O		O			O		O			SigA, SigH
KIAC18_002737	*ftsA*	BSU_15280	24.0	5.96E-18	O	O					O				SigH
KIAC18_003144	*sprC*	BSU_40950	57.2	1.60E-58			O					O			SigF
KIAC18_003351	*prtG*	BSU_00430	42.2	4.96E-77					O	O				O	SigK
KIAC18_003423	*murE*	BSU_15180	28.3	1.89E-53				O					O		SigE
KIAC18_003434	*spo0F*	BSU_37130	51.5	6.85E-34	O	O					O				SigH
KIAC18_003495	*spoVT*	BSU_00560	59.0	1.49E-73			O					O			SigF
KIAC18_003498	*hbs*	BSU_22790	61.8	2.94E-37	O	O					O				SigH
KIAC18_003521	*spcP*	BSU_00600	34.1	1.74E-13				O					O		SigE
KIAC18_003589	*yetF*	BSU_07140	26.4	2.30E-25			O			O		O			SigF
KIAC18_003904	*asnO*	BSU_10790	51.6	0				O					O		SigE

KIAC18_00 4295	*yisY*	BSU_10900	49.1	2.18E-95				O	O	O				O	SigK
KIAC18_00 4390	*pdaB*	BSU_01570	38.7	2.50E-51				O					O		SigE

*Only genes with alternative sigma factor binding motifs in their promoter sequences were considered.

Table S8. Transcription units of hydrogenase genes in KIAC.

Class	Putative Function	Locus tag	Gene	Annotation	TU*
[FeFe]-Hydrogenase					
Group A1 or A4	H_2-dependent CO_2 reductase (HDCR)	KIAC18_000085	*fdhF1*	formate dehydrogenase subunit alpha	TU-0182
		KIAC18_000086	*fdhF2*	formate dehydrogenase subunit alpha	
		KIAC18_000087	*hydB1*	4Fe-4S dicluster domain-containing protein	
		KIAC18_000088	*hydA*	4Fe-4S binding protein	
		KIAC18_000090	*hydB2*	4Fe-4S dicluster domain-containing protein	
Group A1	Cytochrome-containing hydrogenase	KIAC18_001744	*hydH*	cytochrome b/b6 domain-containing protein	TU-1123
		KIAC18_001745	*hydS*	iron hydrogenase small subunit	
		KIAC18_001746	*hydA*	[FeFe] hydrogenase, group A	
Group A3	Electron-bifurcating NAD-reducing hydrogenase (HydABCDE)	KIAC18_000110	*hydC*	NADH-quinone oxidoreductase subunit NuoE	TU-0211
		KIAC18_000111	*hydE*	ATP-binding protein	
		KIAC18_000112	*hydD*	(2Fe-2S) ferredoxin domain-containing protein	
		KIAC18_000113	*hydB*	NADH-quinone oxidoreductase subunit NuoF	
		KIAC18_000114	*hydA*	NADH-dependent [FeFe] hydrogenase, group A6	
	Non-bifurcating NADP-reducing hydrogenase (HydABC)	KIAC18_000072	*hydC*	NAD(P)H-dependent oxidoreductase subunit E	TU-0166**
		KIAC18_000073	*hydB*	NADH-quinone oxidoreductase subunit F	
		KIAC18_000074	*hydA*	NADH-dependent [FeFe] hydrogenase, group A6	
	Non-bifurcating NADP-reducing hydrogenase (HydABC)	KIAC18_003897	*hydC*	NAD(P)H-dependent oxidoreductase subunit E	-
		KIAC18_003898	*hydB*	NADH-quinone oxidoreductase subunit F	
		KIAC18_003899	*hydA*	NADH-dependent [FeFe] hydrogenase, group A6	
	Non-bifurcating NADP-reducing hydrogenase (HydABC)	KIAC18_004086	*hydC*	NAD(P)H-dependent oxidoreductase subunit E	-
		KIAC18_004087	*hydB*	NADH-quinone oxidoreductase subunit F	
		KIAC18_004088	*hydA*	NADH-dependent [FeFe] hydrogenase, group A6	
Group B	Unknown	KIAC18_001102	*Hyd*	iron hydrogenase	-
[NiFe]-Hydrogenase					
Group 1a	Unknown	KIAC18_002723	*hysA*	nickel-dependent hydrogenase large subunit	-
		KIAC18_002724	*hysB*	hydrogenase small subunit	
Group 1d	Cytochrome-containing hydrogenase involved in energy conservation	KIAC18_000217	*hynA*	hydrogenase small subunit	TU-0281
		KIAC18_000218	*hynB*	nickel-dependent hydrogenase large subunit	
		KIAC18_000219	*hynC*	Ni/Fe-hydrogenase, b-type cytochrome subunit	

*If no TUs are detected due to the absence or low expression of hydrogenases, the associated TU is indicated as "-" (none).

**TU-0166 includes 9 genes (*hdrC, hdrB, hdrA, mvhD, metV, metF, hydC, hydB,* and *hydA*). The first six genes were excluded from heterologous expression because MetFV-HdcrABC-MvhD is known to form a large complex.

Table S9. List of oligonucleotides used in this study.

Oligo name	Sequence (5' - 3')	Note
**Purpose: dRNA-seq library amplification indexing primer set**		
PCR1_Universal	AATGATACGGCGACCACCGAGATCTACACTCTTTCCCTACACGACGCTCTTCCGATCT	Forward primer
Index_1	CAAGCAGAAGACGGCATACGAGATCGTGATGTGACTGGAGTTCAGAC	Reverse index primer
Index_2	CAAGCAGAAGACGGCATACGAGATACATCGGTGACTGGAGTTCAGAC	
Index_3	CAAGCAGAAGACGGCATACGAGATGCCTAAGTGACTGGAGTTCAGAC	
Index_4	CAAGCAGAAGACGGCATACGAGATTGGTCAGTGACTGGAGTTCAGAC	
Index_5	CAAGCAGAAGACGGCATACGAGATCACTGTGTGACTGGAGTTCAGAC	
Index_6	CAAGCAGAAGACGGCATACGAGATATTGGCGTGACTGGAGTTCAGAC	
Index_7	CAAGCAGAAGACGGCATACGAGATGATCTGGTGACTGGAGTTCAGAC	
Index_8	CAAGCAGAAGACGGCATACGAGATTCAAGTGTGACTGGAGTTCAGAC	
Index_9	CAAGCAGAAGACGGCATACGAGATCTGATCGTGACTGGAGTTCAGAC	
Index_10	CAAGCAGAAGACGGCATACGAGATAAGCTAGTGACTGGAGTTCAGAC	
Index_11	CAAGCAGAAGACGGCATACGAGATGTAGCCGTGACTGGAGTTCAGAC	
Index_12	CAAGCAGAAGACGGCATACGAGATTACAAGGTGACTGGAGTTCAGAC	
Index_13	CAAGCAGAAGACGGCATACGAGATTGTTGACTGTGACTGGAGTTCAGAC	
Index_14	CAAGCAGAAGACGGCATACGAGATACGGAACTGTGACTGGAGTTCAGAC	
Index_15	CAAGCAGAAGACGGCATACGAGATTCTGACATGTGACTGGAGTTCAGAC	
Index_16	CAAGCAGAAGACGGCATACGAGATCGGGACGGGTGACTGGAGTTCAGAC	
Index_18	CAAGCAGAAGACGGCATACGAGATGTGCGGACGTGACTGGAGTTCAGAC	
Index_19	CAAGCAGAAGACGGCATACGAGATCGTTTCACGTGACTGGAGTTCAGAC	
Index_20	CAAGCAGAAGACGGCATACGAGATAAGGCCACGTGACTGGAGTTCAGAC	
Index_21	CAAGCAGAAGACGGCATACGAGATTCCGAAACGTGACTGGAGTTCAGAC	
Index_22	CAAGCAGAAGACGGCATACGAGATTACGTACGGTGACTGGAGTTCAGAC	
Index_23	CAAGCAGAAGACGGCATACGAGATATCCACTCGTGACTGGAGTTCAGAC	
Index_25	CAAGCAGAAGACGGCATACGAGATATATCAGTGTGACTGGAGTTCAGAC	
Index_27	CAAGCAGAAGACGGCATACGAGATAAAGGAATGTGACTGGAGTTCAGAC	
**Purpose: Plasmid construction for heterologous expression of hydrogenases from *S. sphaeroides* KIAC**		
PU05660_F	ATGATTACGAATTCGAGCTCGCTACCTGGACATGACCGAG	*E. limosum* native promoter fragment
PU05660_R	CCGATTTCTGCCATGGTACCTTTGTTTATTCCTCCTTGAAACAAG	
PU12780_F	ATGATTACGAATTCGAGCTCGATGGGCAGCTATTTCGAGGA	*E. limosum* native promoter fragment
PU12780_R	CCGATTTCTGCCATGGTACCTGCTCCTCCTATCTTTTTCCTTTTT	
HDCR_F_05660	GAGGAATAAACAAAGGTACCATGTACCTGCTGGTAGAAGATGG	KIAC HDCR fragment
HDCR_R	CGACTCTAGAGGATCCTTGCTACGGATCTTGTGCCGA	
EB_F_05660	GAGGAATAAACAAAGGTACCATGAACAGCGACAAAGAAACT	KIAC EB fragment
EB_R	CGACTCTAGAGGATCCGATCCTTATCCGGGAAAATTAG	
NADP_F_12780	AGATAGGAGGAGCAGGTACCATGTGTTGTTCAAAAGTAACAAAAGATCA	KIAC NADP fragment
NADP_R	CGACTCTAGAGGATCCCATAGCCTGTCACTACTCTACAAA	
**Purpose: qRT-PCR**		
HDCR1_F	AGCGTTTAACCAAACCGCTG	KIAC HDCR (*fdhF1*)
HDCR1_R	TGATGGCTTTCAGCCTGTCG	
HDCR2_F	AGTAAACGCCTTACCAAACCCA	

HDCR2_R	TTTGCCGGCTACATAGTCCA	KIAC HDCR (*fdhF2*)
HDCR3_F	CCCGTGTGGAGGAAGTAACC	KIAC HDCR (*hydB1)*
HDCR3_R	ATCAGTACTTTGTCCCCGGC	
HDCR4_F	TGCAGCGCATACCAGGAATA	KIAC HDCR (*hydA)*
HDCR4_R	ATTATGAGCGGCAAACAGCG	
HDCR5_F	GTACCGTTTGTACCCCGGTT	KIAC HDCR (*hydB2)*
HDCR5_R	GGACAATGGCTTTTTCCGGG	
EB1_F	GGTATTGCAGGAAGCGCAAA	KIAC EB (*hydC)*
EB1_R	ACCGTAAATCTGGCTGACCG	
EB2_F	ACCAGGACTACCCGACGAAT	KIAC EB (*hydE)*
EB2_R	AGTACCTTGTCCCGGTATGGA	
EB3_F	TACTTGTGGTATCGCTGCCG	KIAC EB (*hydD)*
EB3_R	CCGATACAACCTGTTTGGCG	
EB4_F	GGGCCCGCTGGTTATCATTT	KIAC EB (*hydB)*
EB4_R	GCGTTCTACAGTACGCCCT	
EB5_F	AAGCGCGCAAGACAGTAGT	KIAC EB (*hydA)*
EB5_R	TCGGCTGCTATAGTTTGCAGT	
NADP1_F	GGCGAAAACTACGTAGCCGA	KIAC NADP (*hydC)*
NADP1_R	GGGCACCGTACTGAACATGG	
NADP2_F	CTGCTGGAAATCCCCGAGTT	KIAC NADP (*hydB)*
NADP2_R	GAGCGGATCCTGGCCTAAAA	
NADP3_F	TCTGCTACCATCCGGACCTT	KIAC NADP (*hydA)*
NADP3_R	CGTCCCAGACTTCGTTGCTG	
catP_F	ACTCAGTCCAAAGGCTGGAAAA	Thiamphenicol resistance gene (*catP*)
catP_R	CGCCATTCAGAGTTTAGGACG	
hydC_F	ACAGAAGTGTATGGGGTAGCA	*E. limosum* endogenous hydrogenase (*hydC*)
hydC_R	TTACATAGCACGCTGTCCCC	
hydD_F	AAAGGACGTTACCGTTGCCC	*E. limosum* endogenous hydrogenase (*hydD*)
hydD_R	CTGCACTTTTTCCGGTGTCAT	
hydB_F	AGAAAGAGGGCGAACATCCG	*E. limosum* endogenous hydrogenase (*hydB*)
hydB_R	CTTCAGGGTCGATAACGCCG	
hydA_F	TACCAACACACCGAAAGCCA	*E. limosum* endogenous hydrogenase (*hydA*)
hydA_R	TTTTCGCTGCGGATACAGGT	

Table S10. List of acetogenic bacteria with complete genome sequences.

Organism	NCBI RefSeq assembly	Chromosome size
Acetohalobium arabaticum DSM 5501	GCF_000144695.1	2,469,596
Blautia producta DSM 2950	GCF_010669205.1	6,197,116
Blautia hydrogenotrophica DSM 10507	GCF_034356035.1	3,590,609
Acetobacterium wieringae CH1	GCF_023700225.1	3,711,234
Acetobacterium woodii DSM 1030	GCF_000247605.1	4,044,777
Eubacterium limosum ATCC 8486	GCF_000807675.2	4,422,837
Eubacterium callanderi KIST612	GCF_000152245.2	4,316,707
Eubacterium maltosivorans YI	GCF_002441855.2	4,337,501
Clostridium autoethanogenum DSM 10061	GCF_001484725.1	4,352,446
Clostridium ljungdahlii DSM 13528	GCA_000143685.1	4,630,065
Clostridium carboxidivorans P7	GCF_001038625.1	5,732,880
Clostridium drakei SL1	GCF_003096175.1	5,695,241
Clostridium scatologenes ATCC 25775	GCF_000968375.1	5,749,410
Clostridium sp. AWRP	GCF_004006395.2	4,579,117
Clostridium aceticum DSM 1496	GCF_001042715.1	4,201,318
Clostridium formicaceticum DSM 92	GCF_002080475.1	4,586,728
Clostridioides difficile 630	GCF_000932055.2	4,274,782
Terrisporobacter petrolearius JCM 19845	GCF_032598785.1	4,096,312
Terrisporobacter mayombei DSM 6539	GCF_031202285.1	4,034,168
Terrisporobacter glycolicus DSM 1288	GCF_036812735.1	4,039,277
Carboxydothermus hydrogenoformans Z-2901	GCF_000012865.1	2,401,520
Thermoanaerobacter kivui LKT-1	GCA_000763575.1	2,397,824
Thermacetogenium phaeum DSM 12270	GCF_000305935.1	2,939,057
Moorella thermoacetica DSM 2955	GCF_001267435.1	2,623,349
Sporomusa sphaeroides DSM 2875	GCF_001941975.2	4,956,256
Sporomusa ovata DSM 2662	GCF_000445445.2	5,433,971
Sporomusa malonica DSM 5090	GCF_041428715.1	5,312,210
Sporomusa aerivorans DSM 13326	GCF_041428895.1	6,496,238
Sporomusa silvacetica DSM 10669	GCF_002257705.2	6,046,356
Sporomusa rhizae DSM 16652	GCF_041428845.1	5,829,736
Sporomusa termitida DSM 4440	GCF_007641255.1	5,184,890
Sporomusa acidovorans DSM 3132	GCF_002257695.2	5,955,502
Sporomusa sphaeroides KIAC	This study	4,844,680

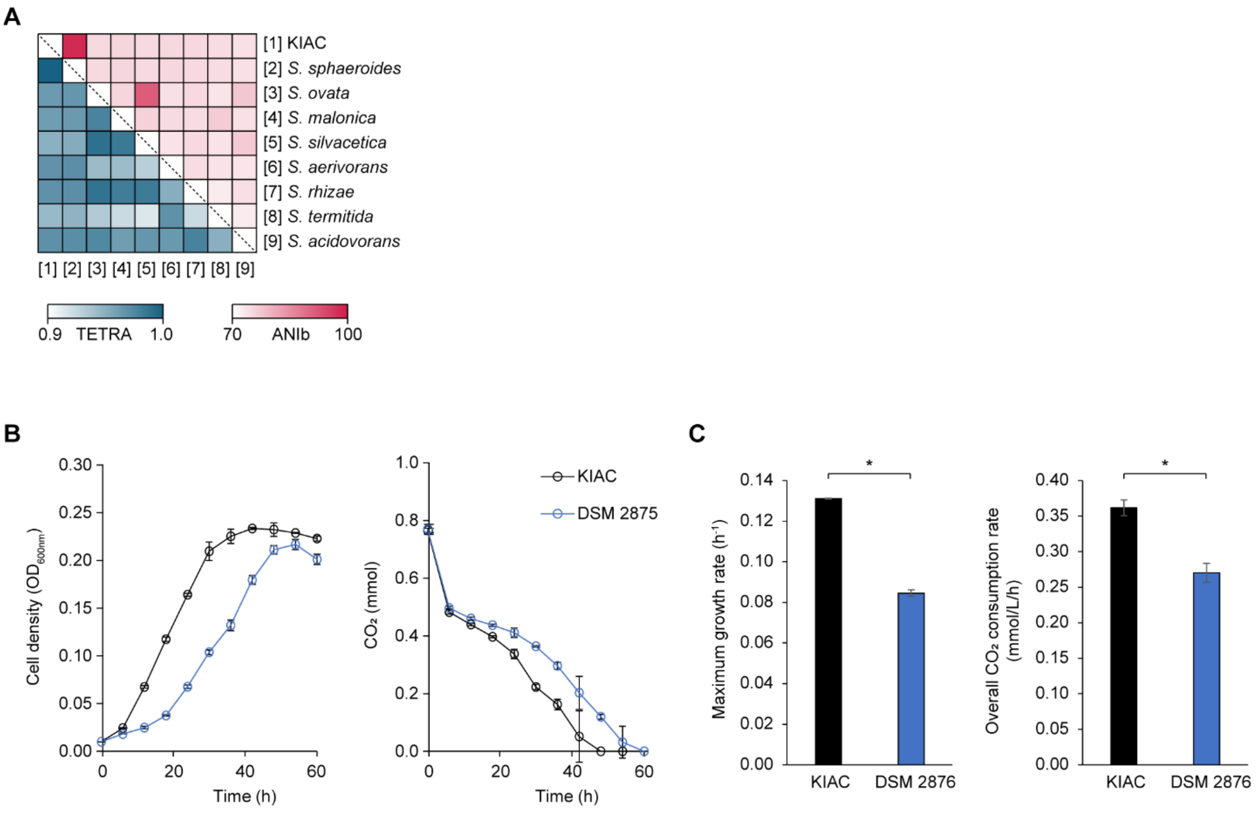

Figure S1. Comparative genomic and phenotypic analysis of *S. sphaeroides* KIAC. (A) Pairwise comparison of ANI based on BLAST (ANIb) and tetranucleotide composition (TETRA) values of KIAC and eight *Sporomusa* species with complete genomes, analyzed via JSpeicesWS (6). Values are shown as a matrix with species ordered identically on both axes. (B) Comparison of growth and CO_2 consumption profiles between *S. sphaeroides* KIAC and *S. sphaeroides* DSM 2875 grown under H_2/CO_2 conditions. (C) Maximum growth rates (h^{-1}) and overall CO_2 consumption rates (mmol L^{-1} h^{-1}) of the two strains were calculated. Statistical significance was assessed using Student's *t* test (*, $P < 0.001$).

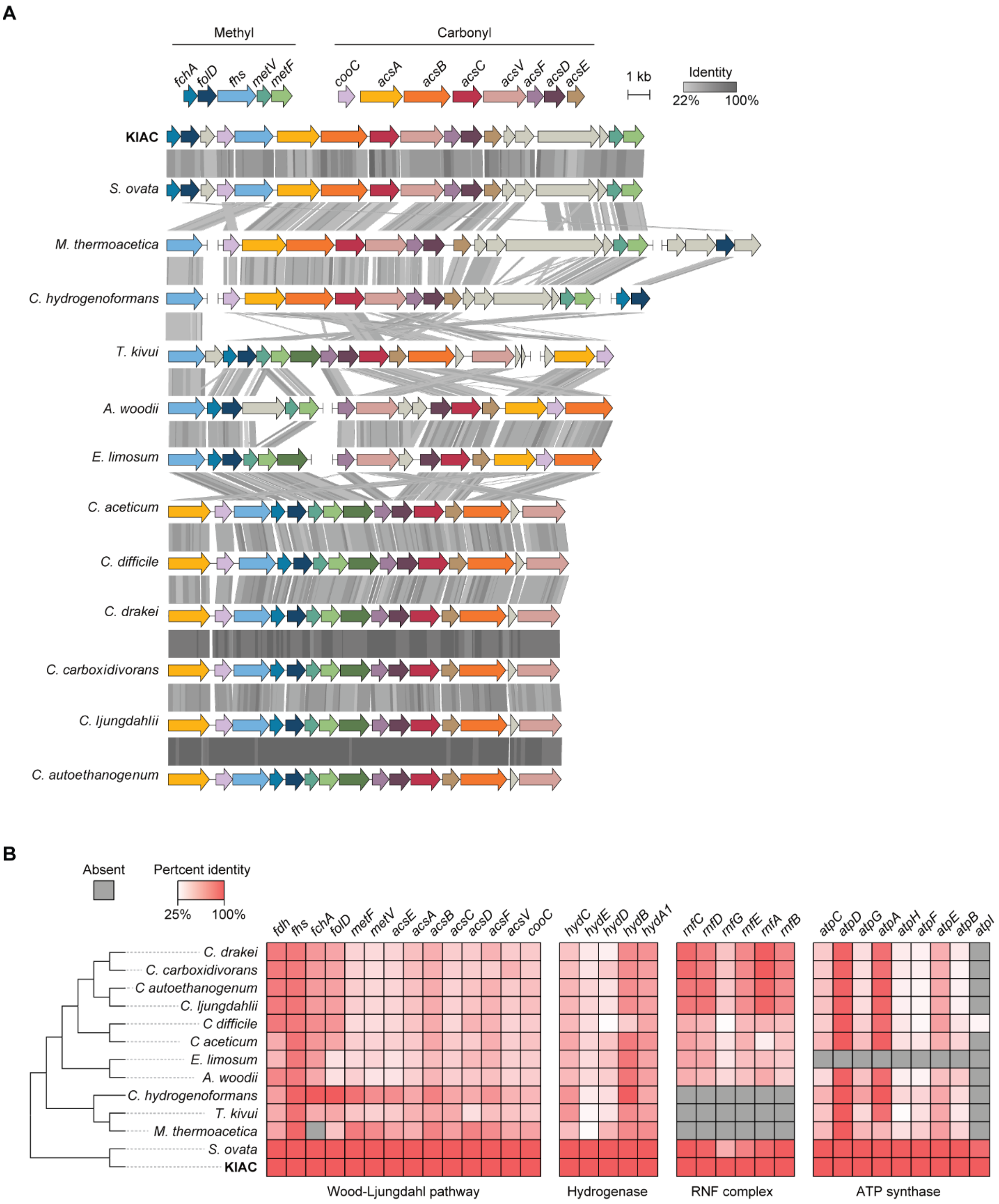

Figure S2. Comparative analysis of acetogenesis-related genes in KIAC. (A) The arrangement of the Wood–Ljungdahl pathway gene cluster across acetogens, including KIAC, visualized with Easyfig (7). Genes are represented by arrows and color-coded according to their function.

Borders between genes or clusters are shown with break lines. (B) Comparison of proteins associated with the Wood-Ljungdahl pathway and energy conservation system between KIAC and 12 acetogenic bacteria. The proteins from KIAC were used as a reference and analyzed using BLAST. The similarity score is colored as indicated on the color key, and the absence of the target protein is colored in dark gray.

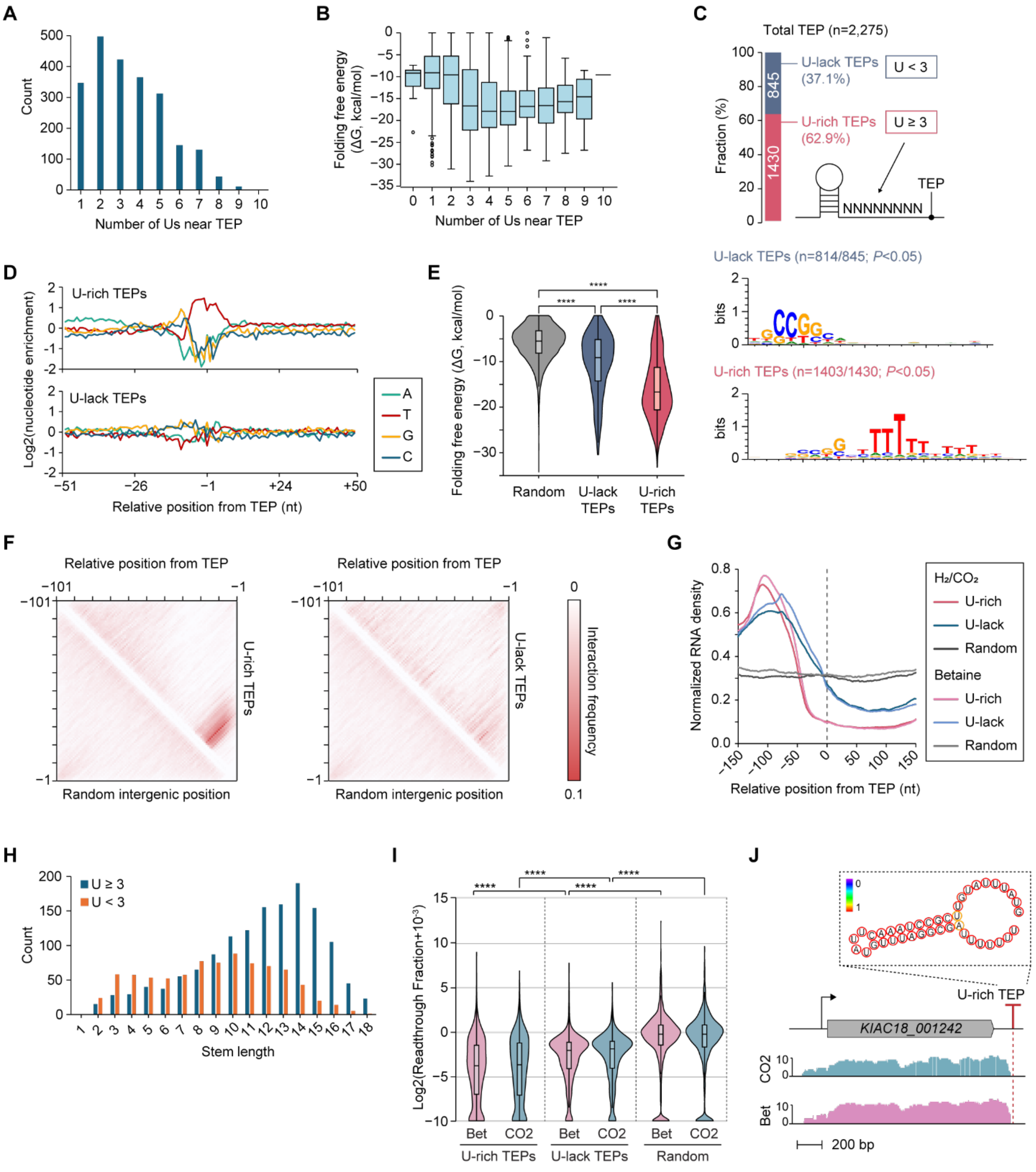

Figure S3. Discovery of two groups of TEPs and their characteristics in KIAC. (A) Distribution of the number of uridines (Us) near TEPs. (B) Folding free energy (ΔG) calculated for each group of TEPs based on the number of Us present near TEPs using RNAfold (8). (C) Schematic representation of the two TEP groups classified based on the number of uridines near TEPs.

Conserved sequence motifs detected by MEME algorithms for each TEP group are shown on the right. Motif detection was performed using sequences spanning 41 nt upstream to 20 nt downstream of each TEP. (D) Nucleotide enrichment at the −50 to +50 nt positions relative to U-rich TEPs (n=1,430) and U-lack TEPs (n=845). (E) Comparison of folding free energies (ΔG) of predicted RNA structures 40 nt upstream sequences of the two TEP groups, calculated using RNAfold. Random represents folding free energies calculated from 10,000 randomly selected intergenic positions. (F) Interaction frequency between two nucleotides located within the 100 nt upstream region of TEPs. U-rich (left, upper triangle) and U-lack (right, upper triangle) TEP groups were compared with random positions (lower triangle in both squares). RNA structures were predicted using the 100 nt upstream sequence of each TEP or randomly selected position with RNAfold at 37 °C. Interaction frequency was calculated as the ratio of observed interactions in the predicted RNA structures to the total entries. (G) RNA-seq read density near the two TEP groups. (H) Distribution of stem lengths for two groups of TEPs, U-rich TEPs (with three or more Us near TEPs) and U-lack TEPs (with fewer than three Us). (I) Distribution of the readthrough fraction, calculated as the average normalized RNA read-count ratio between the −150 to 0 nt region and the 0 to +150 nt region downstream of TEPs and random positions. ****, $P < 0.0001$ (Mann-Whitney U test, two-sided). (J) Predicted secondary structure of one terminator belonging to the U-rich TEP group. The indicated terminator structure includes a stable stem-loop structure followed by a U tract. Log2-scaled RNA levels of the associated gene are shown.

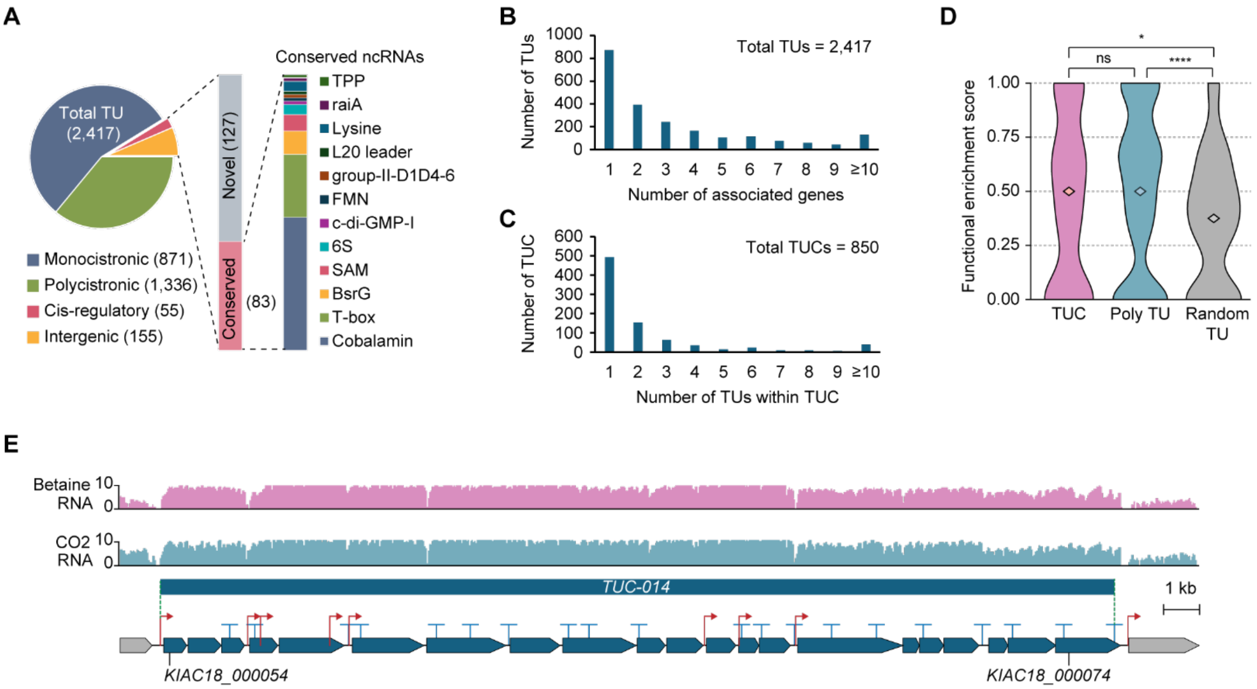

Figure S4. Determination of transcription units (TUs) and TU clusters in KIAC. (A) Categorization of detected TUs and functional prediction of non-coding units using Rfam database (9). (B) Distribution of the number of associated genes within 2,417 TUs. (C) Distribution of the number of associated genes within 850 TUCs. (D) Functional relatedness of genes belonging to same TUC and polycistronic (Poly) TU, compared to randomly selected genes (Random TU). The functional enrichment score was defined as the maximum ratio of the gene number for a single Clusters of Orthologous Groups (COG) functional category in the TU or TUC to the number of total COG categories. Genes without any assigned COG category were excluded. The calculation was performed for TUCs with more than one gene and assigned COG (n=267), polycistronic TUs with more than one gene and assigned COG (n=1025), and for randomly chosen sequential genes on the same strand with assigned COG (n=236). Dots indicate the median, and statistical significance was determined based on P-values (****, $P < 0.0001$; ns, $P > 0.05$; Mann-Whitney U test, two-sided). (E) TUC-014 (KIAC18_000054-000074) containing the WL pathway genes.

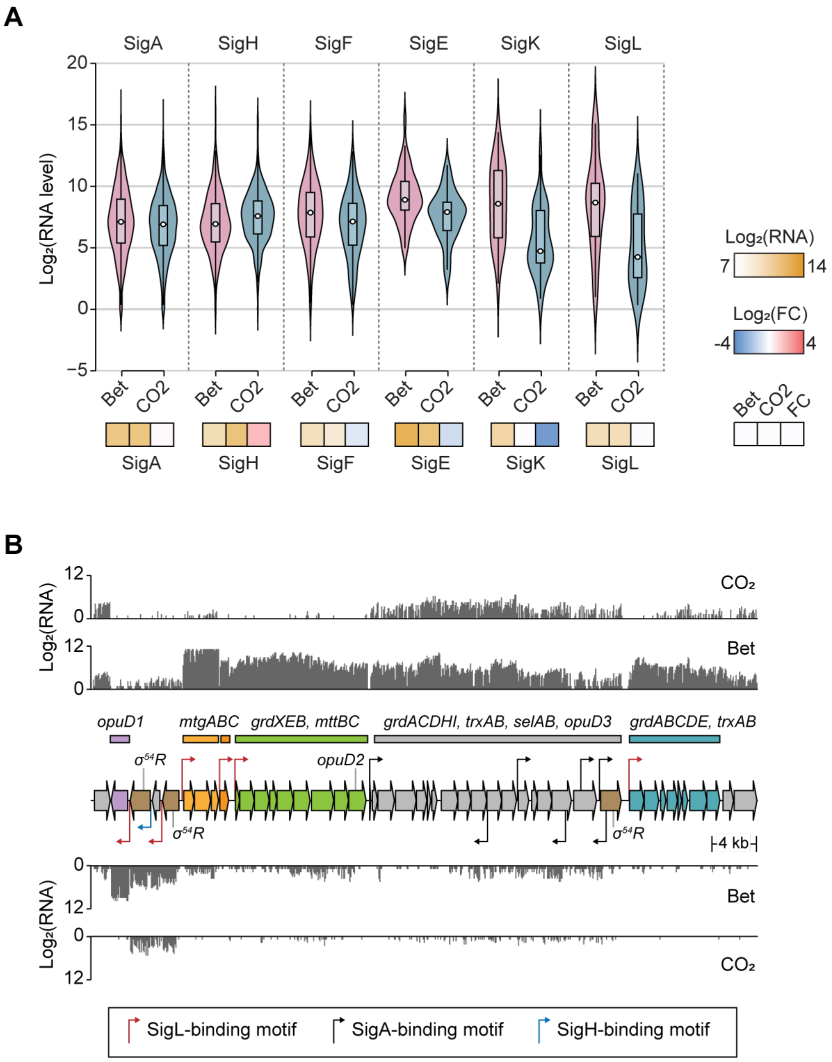

Figure S5. Characteristics of alternative sigma factors and their binding motifs in KIAC. (A) Comparison of RNA transcript levels of genes with each detected sigma factor binding motif in their promoters under betaine (violet) and H_2/CO_2 (blue) conditions. Sigma factor expression levels are shown at the bottom of each plot. (B) A gene cluster associated with betaine metabolism containing genes regulated by SigL with detected SigL binding motifs in their promoters. σ^{54}R represents a σ^{54}-interacting transcriptional regulator.

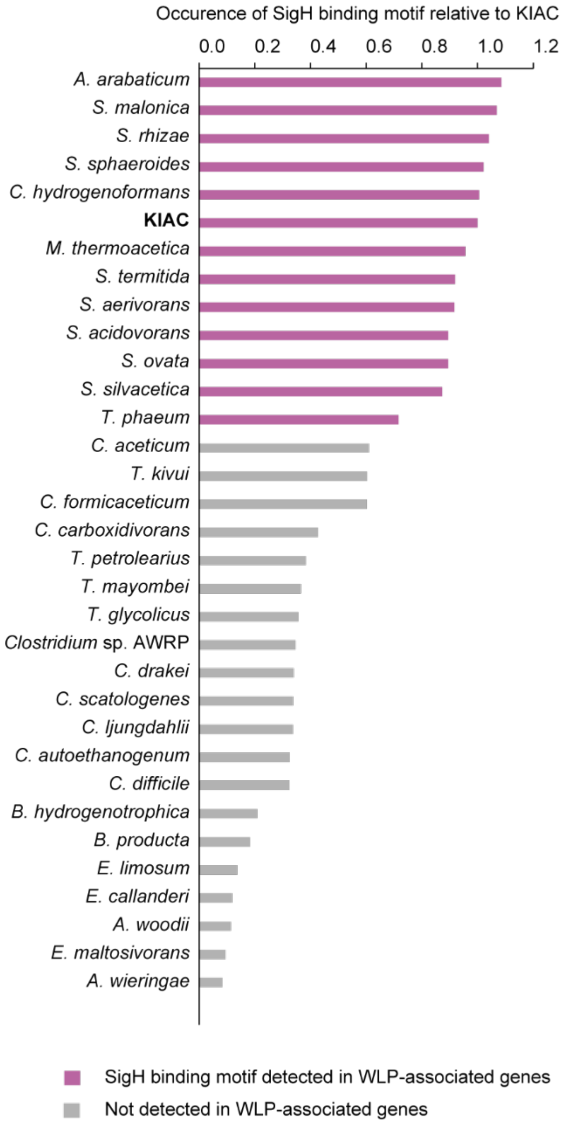

Figure S6. Occurrence of KIAC-like SigH binding motifs within 200-nt upstream sequences of annotated genes across acetogen genomes. Acetogens with SigH binding motifs detected in WL pathway gene clusters are indicated with purple boxes.

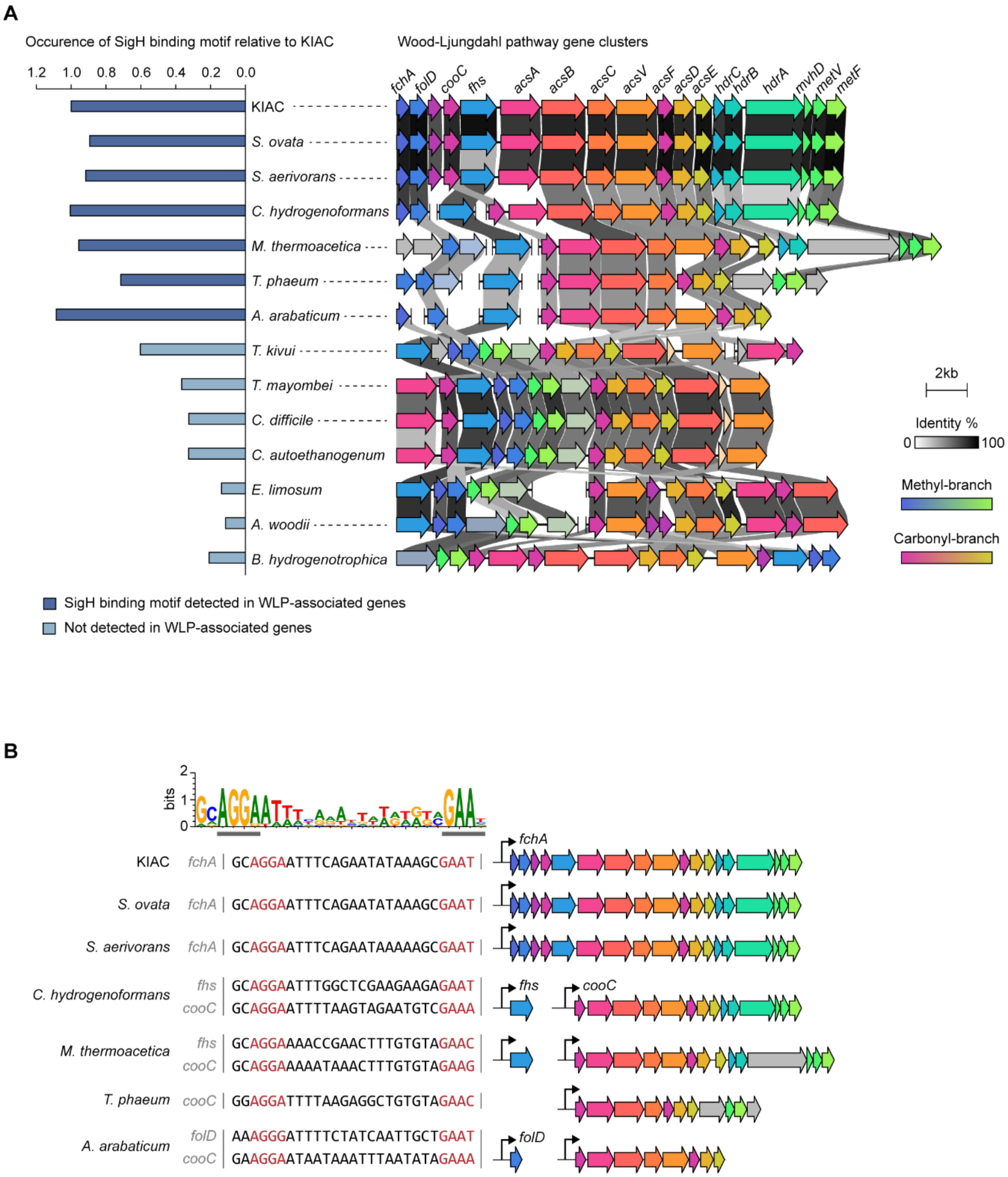

Figure S7. Occurrence of SigH binding motifs across acetogens. (A) Occurrence of SigH binding motifs identified in KIAC within 200-nt upstream sequences of annotated genes in various acetogen genomes. Associated WL pathway gene clusters for each acetogen are also shown. (B) Sequences upstream of annotated genes containing SigH binding motifs in the first

genes associated with the WL pathway gene clusters. Associated WL pathway gene clusters for each acetogen are also shown. In *Sporomusa* species, including KIAC, the motif is present in the first gene (*fchA*) of a single WL pathway gene cluster. In contrast, in *C. hydrogenoformans*, *M. thermoacetica*, *T. phaeum*, and *A. arabaticum*, the motif is detected in *fhs* and *cooC*, which are separated into individual WL pathway gene clusters.

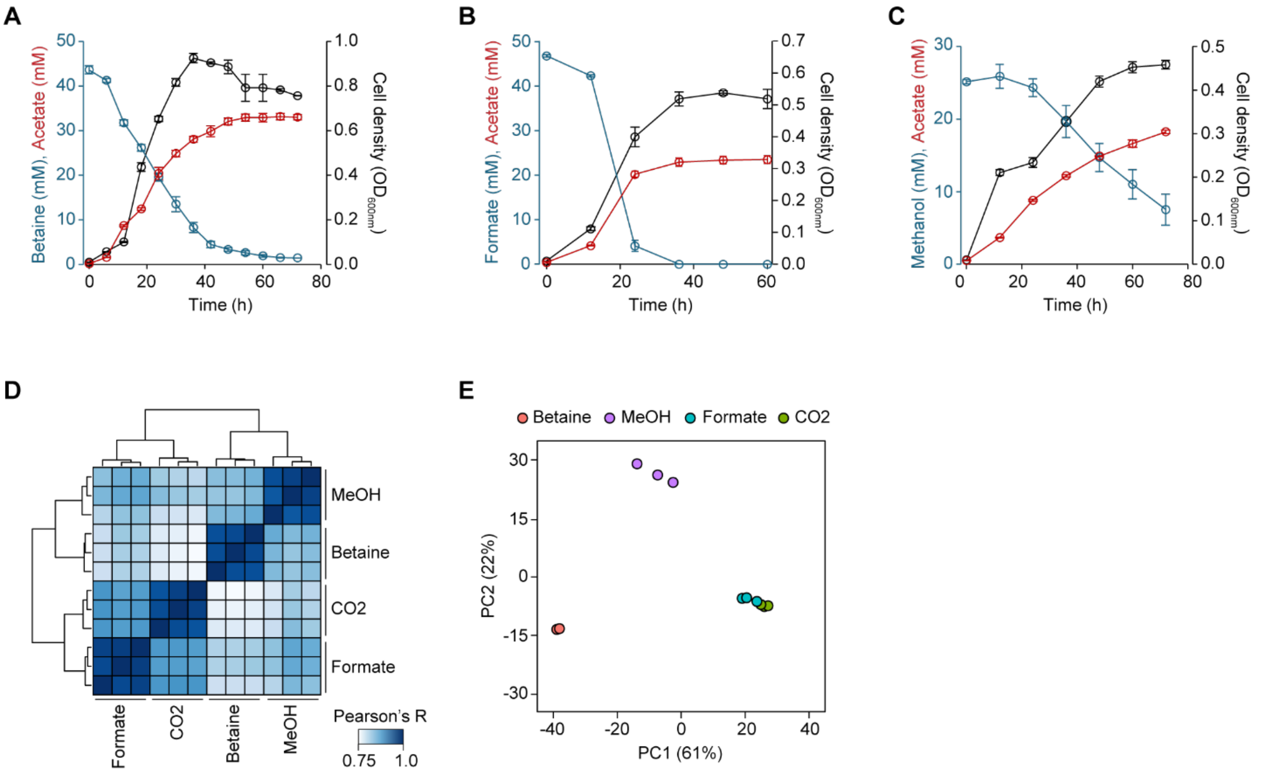

Figure S8. RNA-seq analysis of KIAC under four different growth conditions. Growth curves of *S. sphaeroides* KIAC cultivated under (A) betaine, (B) formate, and (C) methanol. (D) Pearson's correlation coefficient matrix of gene expression profiles from each growth condition, shown with hierarchical clustering using rlog-transformed read counts. Transcriptome patterns formed two distinct clusters: H_2/CO_2 with formate (Pearson's $r > 0.904$) and methanol with betaine (Pearson's $r > 0.871$). (E) Principal components analysis of the RNA-seq data.

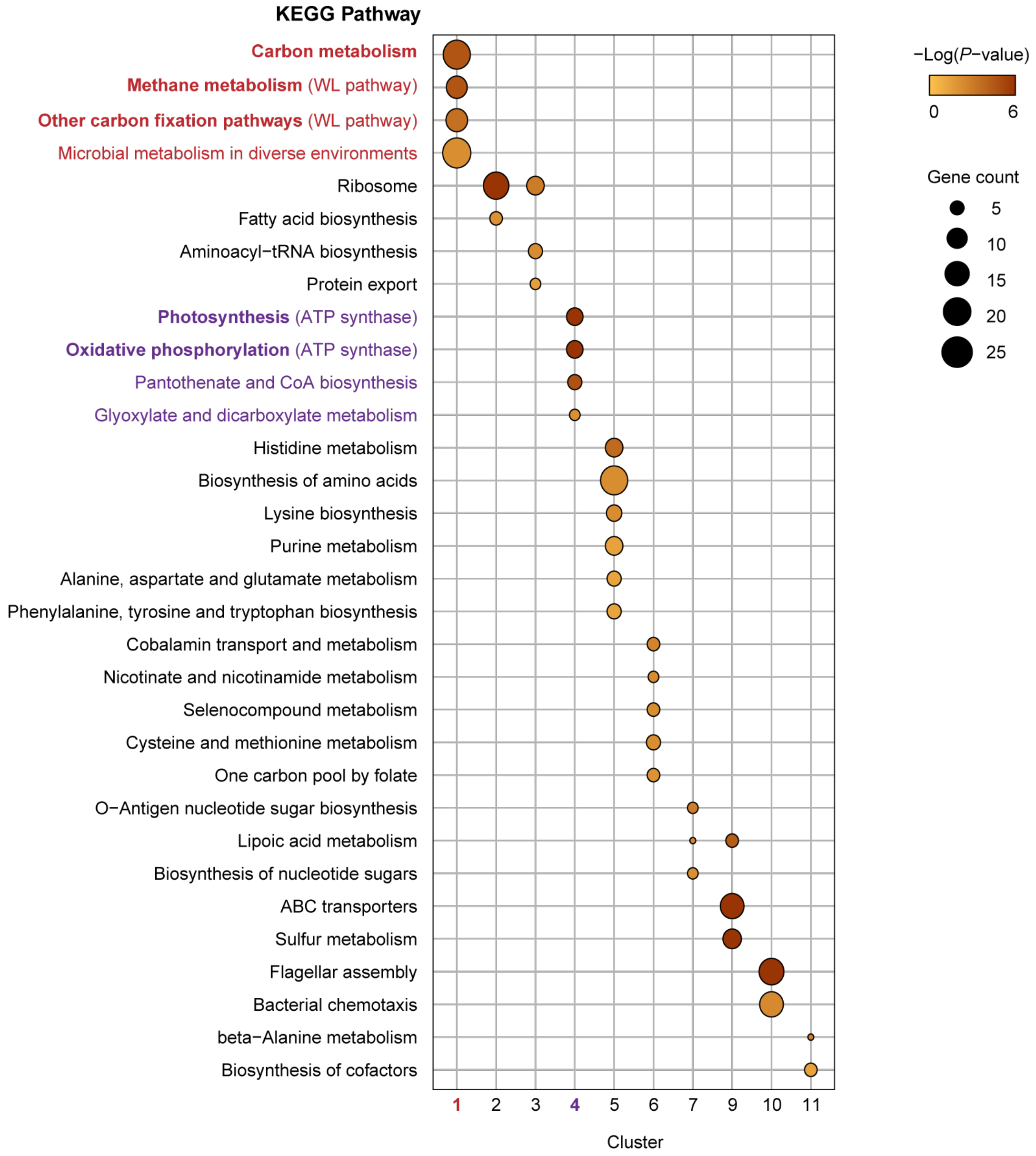

Figure S9. Enriched KEGG pathways for all differentially expressed genes. The KEGG pathway enrichment analysis was performed for 11 groups of clustered genes by ClusterProfiler (10). A Benjamini–Hochberg–corrected $P < 0.05$ was considered significant, and the KEGG pathways enriched in each cluster are shown in $-\log_{10}$ (P-value). KEGG pathways were not enriched for cluster 8. The enriched pathways corresponding to Cluster C1 and Cluster C4 mentioned in the main text are highlighted in red and purple, respectively.

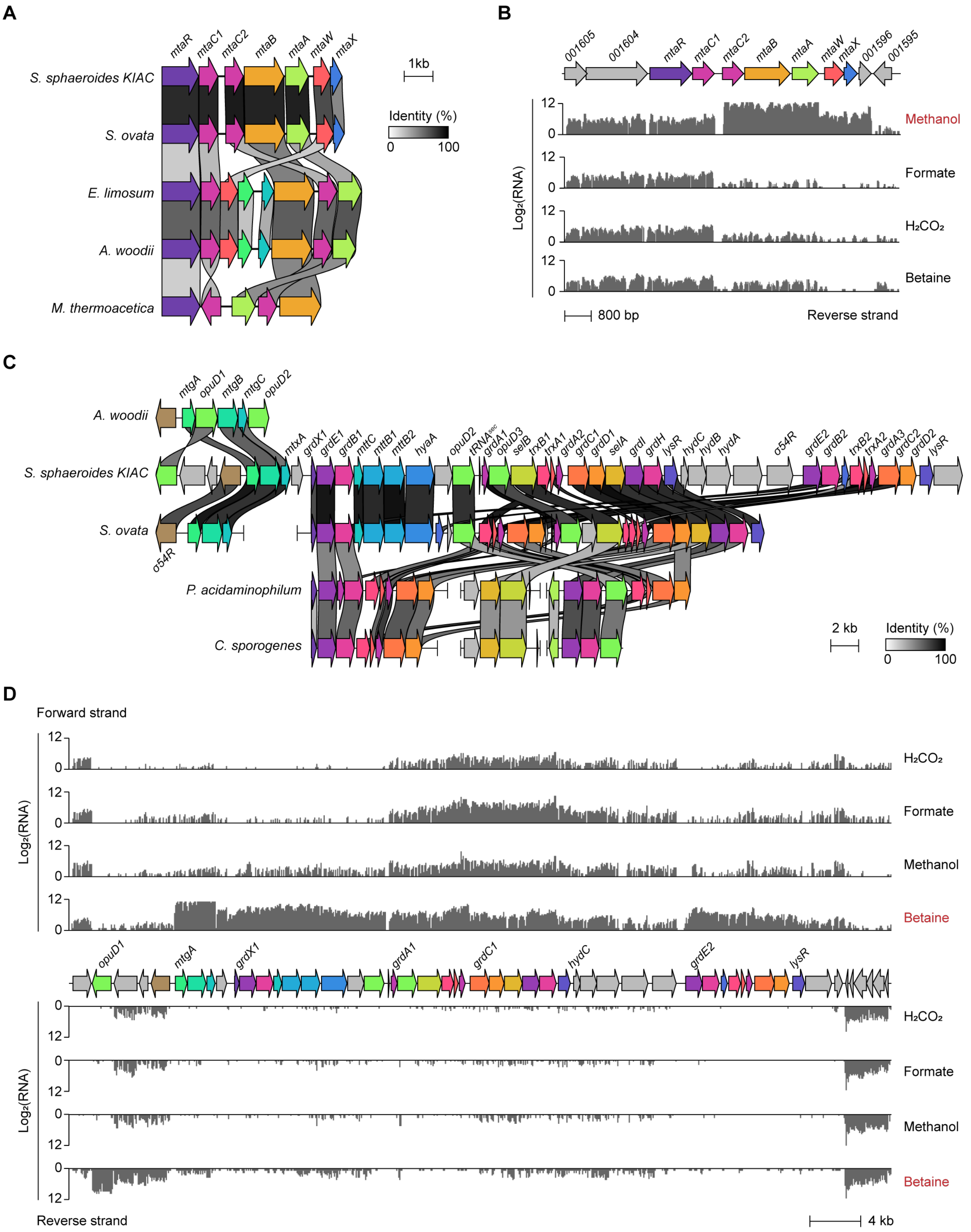
A
mtaR
mtaC1
mtaC2
mtaB
mtaA
mtaW
mtaX
S. sphaeroides KIAC
S. ovata
E. limosum
A. woodii
M. thermoacetica
1kb
Identity (%)
0
100
B
001605
001604
mtaR
mtaC1
mtaC2
mtaB
mtaA
mtaW
mtaX
001596
001595
Log₂(RNA)
12
0
Methanol
Formate
H₂CO₂
Betaine
800 bp
Reverse strand
C
A. woodii
mtgA
opuD1
mtgB
mtgC
opuD2
S. sphaeroides KIAC
S. ovata
σ54R
mtxA
grdX1
grdE1
grdB1
mttC
mttB1
mttB2
hyaA
opuD2
tRNAsec
grdA1
opuD3
selB
trxB1
trxA1
grdA2
grdC1
grdD1
selA
grdI
grdH
lysR
hydC
hydB
hydA
σ54R
grdE2
grdB2
trxB2
trxA2
grdA3
grdC2
grdD2
lysR
P. acidaminophilum
C. sporogenes
2 kb
Identity (%)
0
100
D
Forward strand
Log₂(RNA)
12
0
H₂CO₂
Formate
Methanol
Betaine
opuD1
mtgA
grdX1
grdA1
grdC1
hydC
grdE2
lysR
Reverse strand
4 kb

Figure S10. Alignment of genes involved in methanol and betaine metabolism. (A) Comparison of the *mta* clusters associated with methanol metabolism in *S. ovata* (SOV_RS19095-SOV_RS19065), *Eubacterium limosum* (B2M23_RS16835-RS16870), *Acetobacterium woodii* (AWO_RS11740-RS11705), *Moorella thermoacetica* (MOTHA_RS06205-RS06225), and KIAC (KIAC18_001603-001597). *mtaR* is PocR ligand-binding domain-containing protein; *mtaB* and *mtaA* are methyltransferase; *mtaC1* and *mtaC2* are corrinoid proteins; *mtaW* and *mtaX* are hypothetical proteins. (B) RNA-seq profiles of the *mta* gene cluster present in the genome of KIAC. (C) Comparison of the gene clusters involved in betaine metabolism in *S. ovata*, *Peptoclostridium acidaminophilum, Clostridium sporogenes*, and KIAC (KIAC18_004056-004100). An identity cutoff of 30% was used and visualization was done with the program Clinker (11). Borders between the separate gene clusters are indicated with break lines. (D) RNA-seq profiles of the gene cluster associated with betaine metabolism (~58 kb) present in KIAC. The gene abbreviations are listed in **Data S1**.

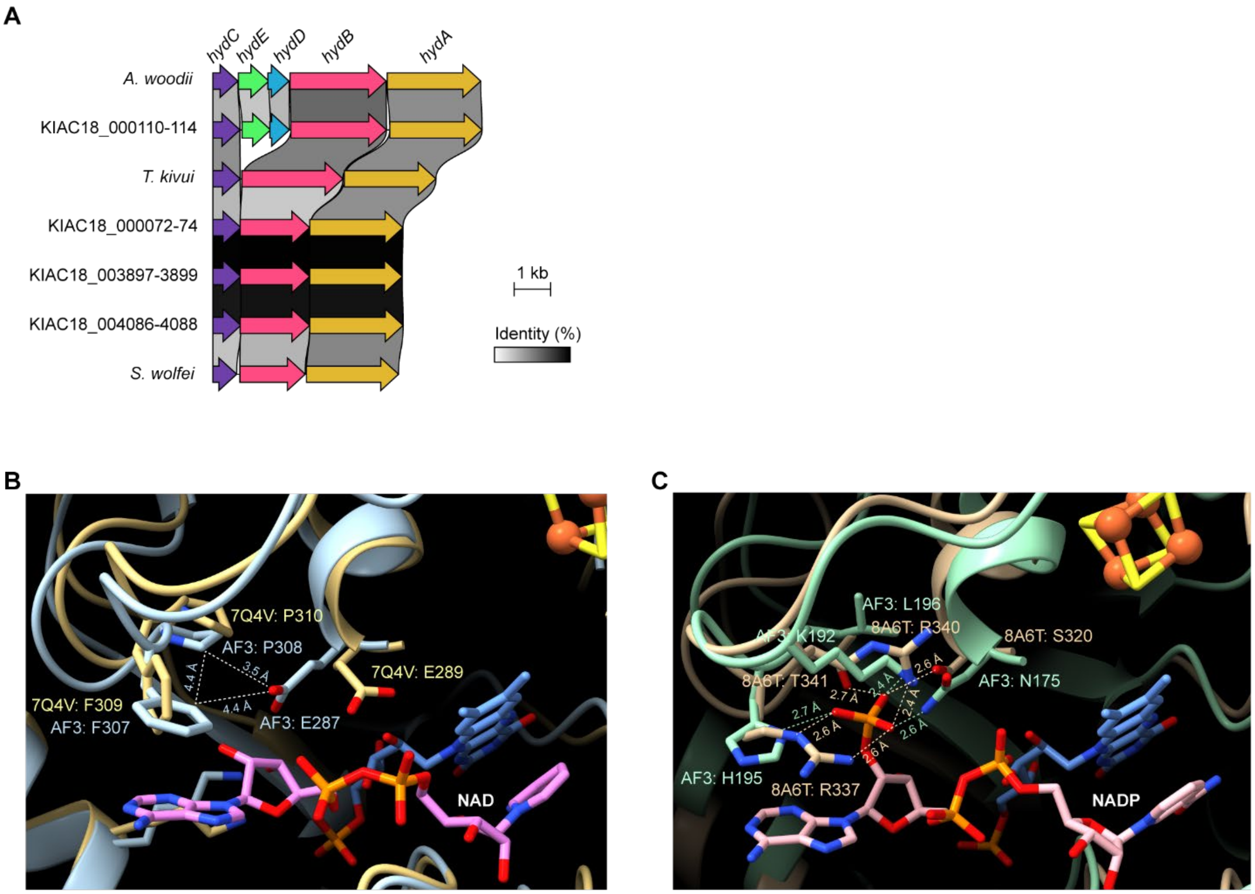

7Q4V: Cryo-EM structure of NAD-binding HydB from *A. woodii*
AF3: AlphaFold3-predicted structure of KIAC18_000113

8A6T: Cryo-EM structure of NADP-binding HydB from *T. kivui*
AF3: AlphaFold3-predicted structure of KIAC18_000073

Figure S11. Comparison of [FeFe]-hydrogenases from KIAC with well-characterized HydABCDE from *A. woodii*, HydABC from *T. kivui*, and HydABC from *S. wolfei*. (A) Alignment of gene clusters of [FeFe]-hydrogenases. Shown is the $NAD(P)^+$ binding site in AlphaFold3-predicted structures of HydB from (B) the putative electron-bifurcating [FeFe]-hydrogenase (KIAC18_000113) and (C) the putative non-bifurcating [FeFe]-hydrogenase (KIAC18_000072). Predicted structures were superimposed onto the cryo-EM structures of NAD-binding HydB from *A. woodii* (PDB: 7Q4V) and NADP-binding HydB from *T. kivui* (PDB: 8A6T), respectively. The NAD/NADP binding sites in the predicted structures were well aligned and consistent with those from *A. woodii* and *T. kivui*, respectively. The phosphate group of NADP interacts with hydrophilic residues located nearby in the range of 2.4~2.7 Å.

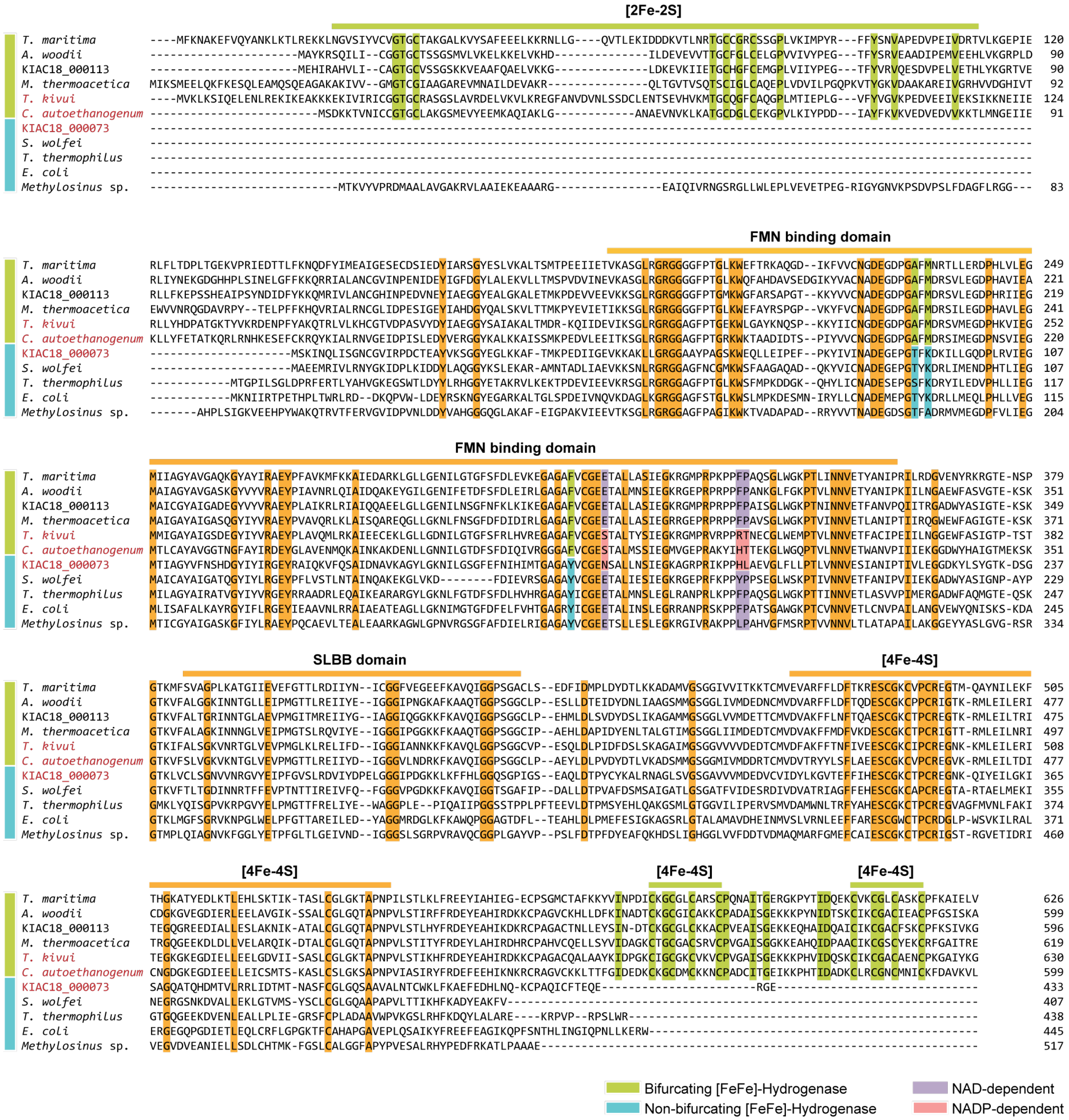

Figure S12. Multiple sequence alignment of HydB. The HydB sequences from Group A3 [FeFe]-hydrogenases present in KIAC, electron bifurcating [FeFe]-hydrogenases, and non-bifurcating [FeFe]-hydrogenases were aligned. Bifurcating hydrogenases from *T. maritima, A. woodii, M. thermoacetica, T. kivui*, and *C. autoethanogenum* were compared and indicated with green boxes. Non-bifurcating hydrogenases from *S. wolfei, T. thermophilus, E. coli,* and

Methylosinus sp. were also compared and indicated with blue boxes. Residues conserved in all hydrogenases are colored orange, conserved in bifurcating hydrogenases are colored in green, and conserved in non-bifurcating hydrogenases are colored in blue. Residues that determine NAD or NADP specificity are colored in purple or pink, respectively.

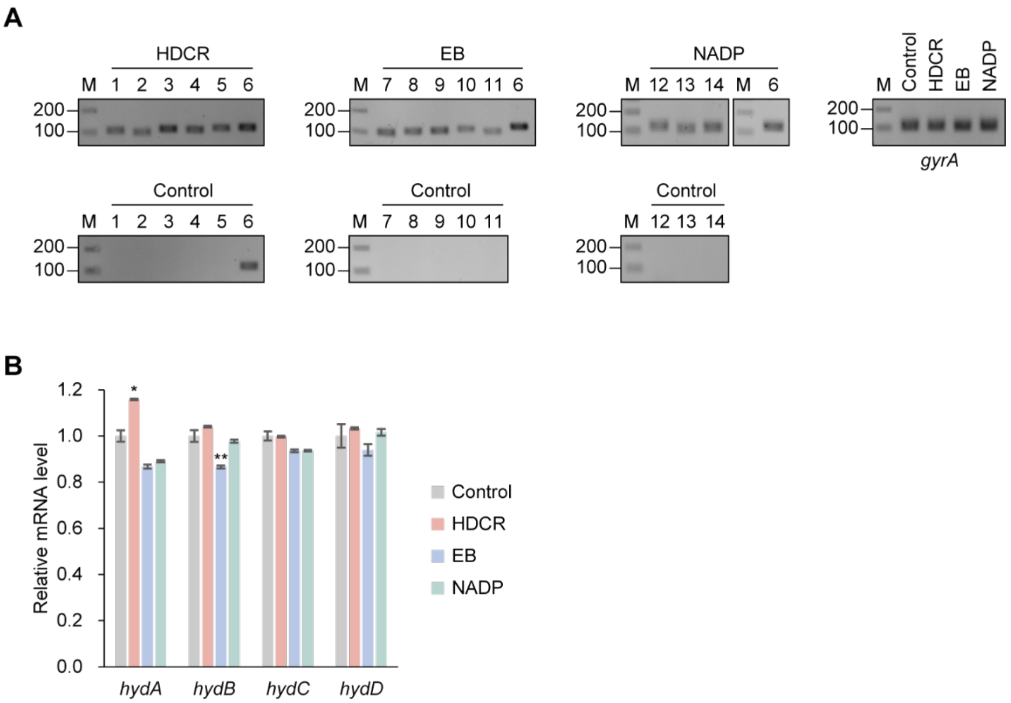

Figure S13. Introduction of hydrogenases from *S. sphaeroides* KIAC into *E. limosum*. (A) Amplicons of hydrogenase gene transcripts from engineered strains grown under autotrophic conditions. The abbreviations for target genes are as follows: HDCR encoding gene (1, *fdhF1*; 2, *fdhF2*; 3, *hydB1*; 4, *hydA*; 5, *hydB2*); thiamphenicol resistance gene present in the plasmid (6, *catP*); electron bifurcating hydrogenase encoding gene (7, *hydC*; 8, *hydE*; 9, *hydD*; 10, *hydB*; 11, *hydA*); non-bifurcating NADP-reducing hydrogenase encoding gene (12, *hydC*; 13, *hydB*; 14, *hydA*); M, size marker; *gyrA*, DNA gyrase subunit A used as a housekeeping reference gene. (B) Quantitative RT–PCR analysis of endogenous *E. limosum* hydrogenase genes in engineered strains. Data are presented as mean ± SEM from three biological replicates. The *gyrA* gene was used as a reference. Statistical significance was assessed using Student's *t* test (**P* < 0.05; ***P* < 0.01).

Supplementary Text

Text S1. Intrinsic termination prevails in Rho-deficient *S. sphaeroides* KIAC

KIAC lacks homologs of the Rho transcription termination factor, consistent with recent findings that 30.4% of Bacillota species lack functional Rho (12). Such Rho-deficient bacteria typically rely on abundant intrinsic terminators (13-15). To examine intrinsic termination in KIAC, we analyzed two key features near TEPs: uridine (U) content and upstream sequence folding free energy (ΔG). TEPs containing three adjacent Us showed substantially lower median ΔG compared to those with one or two Us, indicating their ability to form stable stem-loop hairpin structures characteristic of intrinsic terminators (16) (**Fig. S3A,B**). Based on U content, we classified the 2,275 identified TEPs into U-rich TEPs (62.9%, n = 1,430) and U-lack TEPs (37.1%, n = 845). U-rich TEPs exhibited a characteristic GC-rich region followed by a U-rich tract, while U-lack TEPs displayed only a GC-rich region (**Fig. S3C**).

Nucleotide enrichment analysis of regions spanning −50 to +50 nt relative to TEPs (**Fig. S3D**) revealed that neither group contained sequence features typical of Rho-dependent terminators, such as the downstream C-rich regions characteristic of *E. coli* (17). Instead, both groups showed features of intrinsic terminators. U-rich TEPs contained GC-rich stem-loops (−30 to −10), U-rich tracts (−6 to +6), and A-tracts (−50 to −31). While A tracts of KIAC were longer than those in *E. coli*, their sequence features remained conserved, confirming U-rich TEPs as legitimate intrinsic terminators. U-lack TEPs, though lacking U-rich tracts and A tracts, contained GC-rich stem-loops (−30 to −6). Their median ΔG (−9.4 kcal/mol) was significantly lower than that of random intergenic positions (−5.7 kcal/mol), indicating their capacity to form hairpin structures (**Fig. S3E**). Base-pair interaction analysis of the 100 nt upstream of the TEPs

showed weaker interactions in U-lack TEPs compared to U-rich TEPs (**Fig. S3F**). Despite lacking U-tracts, U-lack TEPs effectively terminated transcription, as evidenced by the gradual decrease in RNA abundance from −100 to −1 under H_2/CO_2 and betaine conditions (**Fig. S3G**).

U-rich TEPs demonstrated superior termination efficiency, exhibiting significantly lower median ΔG (−17.1 kcal/mol) compared to both U-lack TEPs and random positions (**Fig. S9E**), along with longer stem lengths (**Fig. S3H**). These structural features enable robust stem-loop formation and efficient termination, as confirmed by low readthrough fractions and depleted downstream RNA signals (**Fig. S3G, I**). One representative U-rich TEP exemplified these characteristics, showing a conserved GC-rich stem sequence followed by consecutive Us (**Fig. S3J**). While U-lack TEPs function as intrinsic terminators, they demonstrate comparatively weaker termination efficiency. These findings establish that KIAC relies primarily on intrinsic terminators, particularly U-rich TEPs, to define transcript 3′ boundaries in the absence of Rho.

Text S2. Transcription unit determination in KIAC

Integration of TSS, TEP, and RNA-seq data revealed 2,417 putative transcription units (TUs) across both H_2/CO_2 and betaine conditions, comprising 871 monocistronic, 1,336 polycistronic, 55 *cis*-regulatory, and 155 intergenic units (**Data S2** and **Fig. S4A**). The average polycistronic units contained 3.2 genes (**Fig. S4B**), comparable to *E. coli* and *B. subtilis* (18). Overlapping TUs, representing isoforms of longer TUs, were grouped into 850 transcription unit clusters (TUC) (**Data S2** and **Fig. S4C**). Clusters of Orthologous Groups (COG) analysis confirmed functional relatedness of genes within TU clusters and polycistronic units (**Fig. S4D**). Notably, WL pathway genes formed a single TUC-014 (KIAC18_000054-KIAC18_000074) with multiple TU isoforms (**Fig. S4E**).

Analysis of non-coding units using Rfam database (9) identified 83 conserved ncRNAs across 12 families, including various riboswitches and T-box leaders (**Table S5**). Cobalamin riboswitches were the most abundant, controlling vitamin B12 biosynthesis and transport related genes. Other riboswitches included S-adenosyl methionine (SAM) and flavin mononucleotide (FAM) riboswitches regulating methionine and riboflavin biosynthesis, respectively. T-box leaders appeared upstream of amino acid biosynthesis and tRNA ligase genes.

Regarding transcription unit determination, we acknowledge the limitation of inferring transcription units from TSS, TEP, and RNA-seq data, as they may not fully resolve overlapping transcripts or alternative isoforms. Future studies using full-length transcriptome mapping technologies, such as SEnd-seq (19), will be crucial for precisely defining transcription units in acetogens.

Supplementary Methods

Method S1. Enrichment and isolation of acetogenic bacteria

Anaerobic samples from livestock manure and wastewater treatment plants were enriched using the AC-B1 medium, prepared as previously described (20). 5% of samples were inoculated into 100-mL serum vials containing 30 mL AC-B1 medium and incubated at 30 °C under H_2/CO_2 (80:20, v/v). After subculturing, the enriched cultures were serially diluted (10^0, 10^{-1}, 10^{-2}, 10^{-3}), streaked onto AC-B1 agar plates, and incubated anaerobically at 30 °C. The resulting individual colonies were subcultured for isolation. *Sporomusa sphaeroides* KIAC has been deposited in the Korean Collection for Type Cultures (KCTC) with the accession number KCTC 19184P.

Method S2. Analytical methods

Substrates and metabolites were measured using high-performance liquid chromatography (Shimadzu) equipped with a refractive index detector and MetaCarb 87 H 300 × 7.8 mm column (Agilent Technologies) operated at 37 °C with 0.007 N H_2SO_4 as the mobile phase (0.6 mL/min). Glycine betaine was analyzed with a Primesep® 100 (150 × 4.6 mm, 5 µm) column (SiELC Technologies) using 10 % acetonitrile + 0.5 % phosphoric acid as the mobile phase at 0.6 mL/min and 40 °C. Gaseous substrates were measured via gas chromatography (Shimadzu) using a thermal conductivity detector and ShinCarbon ST Micropacked column (Restek) with helium or nitrogen as carrier gas (30 mL/min). The oven temperature was initially set at 30 °C, increasing at 5 °C/min to 100 °C. Injector and detector temperatures were set to 100 °C. The ^{13}C content of acetate was analyzed using GC-mass spectrometry (Agilent Technologies) with an HP-CHIRAL-20B column (Agilent Technologies) with helium as a carrier gas (2 mL/min). The

oven temperature was initially set at 40 °C for 5 min, then increased at 15 °C/min to 180 °C. Injector and transfer line were set to 235 °C and 230 °C, respectively. The ionization mode was EI (70 ev), detection mode was SCAN (mass range 30-100). Injection volume was set to 1 μL and the injection mode was Split (ratio = 5:1).

Method S3. Sequencing and data analysis

Genome sequencing. The complete genome sequence of *Sporomusa sphaeroides* KIAC was determined using PacBio SMRT and illumina platforms at LabGenomics (Korea). Genomic DNA was extracted using the Qiagen Blood & Cell Culture DNA Mini Kit (Qiagen) following the manufacturer's protocol. For short-read sequencing, DNA libraries were prepared using the Illumina TruSeq DNA PCR-Free Kit and sequenced on an Illumina NovaSeq 6000 (2 × 150 bp). Long-read sequencing was performed using the SMRTbell® Express Template Preparation Kit v2.0 (Pacific Biosciences) and sequenced on a PacBio Sequel II. The PacBio platform generated 1,015,154 subreads with an N50 of 12,227. Reads were assembled de novo using Flye (v2.8.3) (21) and genome circularization was confirmed with Circlator (v1.5.5) (22). *dnaA* gene was repositioned at the genome's start. Gene prediction and annotation were performed using the NCBI PGAP (23). The genome sequence of strain KIAC has been deposited in GenBank under the accession number CP181154.

RNA extraction. Biological triplicates of cell cultures were collected at mid-exponential phase by centrifugation at 6,000 × *g* at 4 °C for 10 min and resuspended in 500 μL lysis buffer (20 mM Tris-HCl pH 7.4, 140 mM NaCl, 5 mM $MgCl_2$, 1% Triton X-100). Resuspended cells were flash-frozen in liquid nitrogen, ground with a mortar and pestle, and centrifuged at 4000 × *g* for 10 min

at 4°C to remove debris. Total RNA was extracted using TRIzol (Thermo Scientific) and treated with DNase I (NEB) to remove residual genomic DNA.

RNA seq library preparation. Ribosomal RNA was depleted from RNA sample using the RiboRid method (24). Custom anti-rRNA oligonucleotide probes hybridized to *S. sphaeroides* KIAC rRNAs, followed by RNase H digestion of RNA:DNA heteroduplexes and DNase I treatment to remove primer dimers. RNA-Seq libraries were prepared from 10–100 ng rRNA-depleted RNA using a TruSeq Stranded mRNA Library Prep Kit (Illumina) and validated with a Qubit 4 fluorometer (Invitrogen) and TapeStation 4150 equipped with a High Sensitivity D1000 Screen Tape (Agilent Technologies).

dRNA-seq library preparation. Total RNA was divided into TEX-treated (TEX+) and non-treated (TEX−) samples. TEX+ samples were incubated with 1 U of Terminator 5′-Phosphate-Dependent Exonuclease (TEX, Epicentre) in Terminator Reaction Buffer A (Epicentre) with 20 U of SUPERase In RNase Inhibitor (Thermo Scientific) to enrich primary transcripts, while TEX− samples remained untreated in the same reaction mixture. After reaction termination, RNA was purified using RNA Clean & Concentrator-5 Kit (Zymo). To ligate 5′-RNA adaptor, 5′ triphosphate was converted to monophosphate using 20 U of RNA 5′-polyphosphatase (Epicentre) with 20 U of SUPERase In RNase Inhibitor, followed by incubation at 37 °C for 1 h. After RNA purification using RNA Clean & Concentrator-5 Kit, 5 pmol of 5′-RNA adaptor (5′-ACACUCUUUCCCUACACGACGCUCUUCCGAUCU-3′) was ligated using T4 RNA Ligase 1 (NEB) at 23 °C for 2 h 30 min in a reaction mixture containing 2 μL of 10× T4 RNA ligase 1 buffer, 2 μL of 10 mM ATP, 2 μL DMSO, 8 μL of 50% PEG8000, 2 μL T4 RNA Ligase 1, and 1 μL of SUPERase In RNase Inhibitor (20 U/μL). To prevent unintended 3′ end ligation, the adaptor was dephosphorylated with FastAP Thermosensitive Alkaline Phosphatase (Thermo) to

remove 5′ phosphate groups before ligation. Adaptor-ligated RNA was then subjected to rRNA depletion using RiboRid (24). cDNA was synthesized using random hexamer 3′ overhanging primer (5′-GTGACTGGAGTTCAGACGTGTGCTCTTCCGATCTNNNNNN-3′) and SuperScript III First-Strand Synthesis System (Invitrogen). cDNA libraries were purified using 0.8× volume of Agencourt AMPure XP Beads (Beckman Coulter) and amplified using Phusion High-Fidelity polymerase (Thermo) with indexed primers listed in **Table S9**. Amplification was monitored on the Applied Biosystems^{TM} StepOne^{TM} Real-Time PCR System (Thermo) and stopped before reaching the plateau. The final dRNA-seq libraries were purified with 0.8× AMPure XP beads and validated using Qubit 4 fluorometer and 4150 TapeStation System.

Term-seq library preparation. Term-seq libraries were constructed with modifications to a published protocol (24, 25). Briefly, 5′-DNA adaptor was ligated to RNA using T4 RNA Ligase 1 (NEB) with 150 pmol amino-blocked 3′-DNA adaptor (5′-p-NNAGATCGGAAGAGCGTCGTGTAGGGAAAGAGTGT-AmMO-3′), followed by RNA purification. The adaptor-ligated RNA was then subjected to rRNA depletion via RiboRid (24). and fragmented at 72 °C for 90 s using RNA Fragmentation Reagent (Ambion), followed by purification with 2.2× AMPure XP Beads. cDNA was synthesized using 10 pmol reverse transcription primer (5′-TCTACACTCTTTCCCTACACG-3′) and SuperScript III First-Strand Synthesis System (Invitrogen). After purification using AMPure XP Beads, 150 pmol cDNA 3′-adaptor (5′-p-NNAGATCGGAAGAGCACACGTCTGAACTCCAGTCAC-AmMO-3′) was ligated using T4 RNA Ligase 1 (NEB) at 23 °C for 8 h, followed by purification using 1.8× AMPure XP Beads. The ligation product was amplified using Phusion High-Fidelity DNA Polymerase (Thermo) with indexed primers (**Table S9**) and stopped before reaching the plateau.

The final library was purified using 0.8× AMPure XP beads and validated using Qubit 4 fluorometer and 4150 TapeStation System.

High-throughput sequencing. RNA-seq libraries were sequenced on an Illumina MiSeq (2 × 75 bp), while dRNA-seq and Term-seq libraries were sequenced on an Illumina NextSeq 1000 (1 × 100 bp). Each library generated 6.5–31.1 million reads. Sequencing data were processed and mapped to the KIAC genome using CLC Genomics Workbench 6.5.1 (Qiagen), yielding 75.6–96.9% uniquely mapped reads with 94.3–510.9-fold genomic coverage (**Table S3**). rRNA contamination was below 1%, confirming efficient rRNA depletion.

RNA-seq data processing and transcriptome analysis. Low-quality reads were trimmed (quality limit, 0.05; maximum ambiguous nucleotides, 2), and mapped to the reference genome (mismatch cost, 2; insertion cost, 3; deletion cost, 3; length fraction, 0.9; similarity cost, 0.9). Uniquely mapped reads were retrieved and then normalized using the Bioconductor package DEseq2 with default parameters (26). A total of 2,059 differentially expressed genes (DEGs) were identified ($\log_2$ |fold change| > 1, adjusted P-value < 0.01) across at least two conditions. DEGs were initially grouped into 20 clusters based on error sum of squares (SSE) analysis using unsupervised k-means clustering and further categorized into 11 clusters based on condition-specific expression patterns. Functional enrichment analysis of DEGs was conducted using KEGG Orthology (blastKOALA (27)) annotations. KEGG pathway enrichment analysis was performed using ClusterProfiler (10), and a Benjamini–Hochberg–corrected $P < 0.05$ was considered significant.

dRNA-seq and Term-seq data processing. Low-quality reads and adaptor sequences were trimmed from reads. Additionally, for dRNA-seq, reads < 25 nt after trimming were discarded.

For Term-seq, two random nucleotides added during adaptor ligation were removed from both ends and reads < 15 nt were discarded. Term-seq reads were inverted, as the sequencing was generated in the reverse direction. Finally, resulting trimmed reads were mapped to the reference genome (mismatch cost, 2; insertion cost, 3; deletion cost, 3; length fraction, 0.9; similarity cost, 0.9; and ignore nonspecific match), and only uniquely mapped reads were retained.

Method S4. Identification of TSSs, TEPs, motifs, and transcription units

Identification of transcription start sites. The 5′-end positions of uniquely mapped dRNA-seq reads from TEX+ libraries were considered potential TSSs. TSSs were identified and curated by comparing TEX+ and TEX− datasets, as described previously with slight modifications (28). Briefly, initial TSS peaks within 100 nt were clustered, and adjacent peaks in each cluster were sub-clustered based on a standard deviation (< 10) to select a local maximum peak as the TSS in each sub-cluster. Clusters with ≥3 read counts were considered, and the TSS peaks with maximum read counts were selected as potential TSSs. To refine TSS selection, assigned TSSs were compared with TEX− data, retaining only those present within ±5 nt. Final TSSs were manually inspected against RNA-seq profiles. TSSs were categorized based on their genomic positions relative to annotated genes. Among the TSSs located from 300 nt upstream to 100 nt downstream of the 5′-end of respective gene, those with the highest peak intensities (read counts) were classified as primary (P), while the others as secondary (S) TSSs. TSSs located within annotated genes or on the opposite strand were classified as internal (I) or antisense (A), respectively. TSSs that did not fall into either of these categories were classified as intergenic (N). The TSS results obtained from two conditional libraries were merged within ±5 nt and

designated as total TSSs, which are listed in **Data S2**. Data visualization was performed using SignalMap (Roche NimbleGen, Inc.).

Identification of transcript 3′-end positions. The 3′-end positions of uniquely mapped Term-seq reads were considered as potential TEPs and identified using a combined method from previous studies (29, 30). Initial peaks were clustered as described for TSS peak clustering. Low-intensity peaks <10 read counts (90th percentile) and peaks absent in all three biological replicates were discarded. Further filtering was applied based on peak enrichment, selecting peaks with a z-score above 6. Only reproducible peaks with the highest read count across replicates were retained as TEPs, followed by manually inspected against RNA-seq profiles. TEPs were classified based on genomic positions relative to annotated genes. Among the TEPs located less than 300 nt downstream of the respective gene, TEPs with the highest read counts were classified as primary (P), while the others as secondary (S) TEPs. TEPs located within annotated genes or on the opposite strand were classified as internal (I) or antisense (A), respectively. TEPs that did not fall into either of these categories were classified as intergenic (N). For TEPs located between 100 nt downstream of the primary TSS and the 5′-end of the associated gene, they were classified as *cis*-regulatory (C) TEPs. The minimum distance was set to 100-nt to ensure proper formation of terminator structure. The TEP results obtained from two conditional libraries were merged within ±5 nt and designated as total TEPs, which are listed in **Data S2**.

Motif detection. To identify conserved motifs within promoter regions, sequences 40 nt or 50 nt upstream of identified TSSs were extracted and analyzed by MEME with zoops mode (31). Results from both sequence sets were merged, removing redundancies. For SigH binding motifs, motif refinement was performed due to variations in spacer lengths (17 or 18 nt) between −10

and −35 elements, which can obscure consensus motifs. To address this, 20 nt upstream and 20−40 nt upstream of TSSs initially detected with SigH motifs were separately analyzed using MEME with oops mode to identify −10 and −35 elements. Only sequences with $P < 0.05$ were retained. The final σ-factor binding motifs were visualized using Weblogo (32). For RBS motifs, 20 nt upstream of start codons in genes with 5′-UTR lengths ≥10 nt were analyzed. For terminator motifs, sequences from 41 nt upstream to 20 nt downstream of each TEP were aligned and analyzed using MEME with oops mode. For SigH binding motif searches in acetogen genomes, the SigH motif identified in KIAC was converted into a position-specific probability matrix (PSPM) and scanned across acetogen genomes using FIMO ($P < 1e{-5}$) within MEME suite (33), searching up to 200 nt upstream of annotated genes. Occurrence in each genome was normalized relative to KIAC by the number of annotated genes.

Determination of transcription units. TUs were determined as previously described (30) by integrating TSS and TEP data with RNA-seq profiles. A TU was defined as the connected region between a TSS and a TEP, and those identified in both conditions were merged within a ±5 nt range. Total TUs are listed in **Data S2**. TUs were categorized into coding TUs (monocistronic or polycistronic) and non-coding TUs (cis-regulatory or intergenic). The functions of non-coding TUs were predicted using the Rfam database (9), with identified ncRNAs listed in **Table S5**. TU clusters were defined as a group of overlapping TUs sharing at least 1-nt.

Method S5. Heterologous expression of KIAC hydrogenases in *E. limosum*

Determination of growth rates, substrate uptake rates, and product secretion rates. Growth rates and uptake/secretion rates were determined from measured biomass (OD_{600nm}) and metabolite concentrations, as previously described (34). Rates were calculated individually for

each replicate using regression analysis of measurements collected during the exponential growth phase of batch cultures. Mean values and standard deviations were then determined. The growth rate (μ) was obtained by plotting the natural logarithm of OD_{600nm} values against cultivation time, with the slope of the linear regression representing μ. Mean values and standard deviations were subsequently determined. Biomass-specific substrate uptake and product secretion rates (mmol OD_{600}^{-1} h^{-1}) were calculated by normalizing to OD_{600} values rather than gram dry cell weight (gDW). This approach was necessary because direct measurement of gDW was technically infeasible under H_2/CO_2 conditions due to the extremely low biomass levels. Therefore, OD_{600}-based normalization was used to enable consistent and comparative analysis across strains under these conditions.

Quantitative RT-PCR. Total RNA was extracted from biological triplicates of 100 mL cultures at mid-exponential phase by centrifugation (12,000 × g, 4 °C, 15 min). Genomic DNA was removed using RNase-free DNase I (NEB). cDNA synthesis was performed using the Superscript III First-Strand Synthesis System (Invitrogen). Quantitative real-time PCR was conducted using SYBR FAST qPCR master mix (Kapa Biosystems) with primers listed in **Table S9** on an Applied Biosystems™ StepOne™ Real-Time PCR System (Thermo). Primer specificity was confirmed by melt curve analysis and electrophoresis on 2% agarose gels. Relative mRNA levels were normalized to *gyrA* (DNA gyrase subunit A) as a reference.

References

1. Bertsch J, Müller V. 2015. CO metabolism in the acetogen Acetobacterium woodii. Applied and environmental microbiology 81:5949-5956.
2. Shin J, Song Y, Kang S, Jin S, Lee J-K, Kim DR, Cho S, Müller V, Cho B-K. 2021. Genome-scale analysis of Acetobacterium woodii identifies translational regulation of acetogenesis. Msystems 6:10.1128/msystems. 00696-21.
3. Song Y, Lee JS, Shin J, Lee GM, Jin S, Kang S, Lee J-K, Kim DR, Lee EY, Kim SC. 2020. Functional cooperation of the glycine synthase-reductase and Wood–Ljungdahl pathways for autotrophic growth of Clostridium drakei. Proceedings of the National Academy of Sciences 117:7516-7523.
4. Litty D, Müller V. 2021. Butyrate production in the acetogen Eubacterium limosum is dependent on the carbon and energy source. Microbial Biotechnology 14:2686-2692.
5. Aryal N, Tremblay P-L, Lizak DM, Zhang T. 2017. Performance of different Sporomusa species for the microbial electrosynthesis of acetate from carbon dioxide. Bioresource technology 233:184-190.
6. Richter M, Rossello-Mora R, Oliver Glockner F, Peplies J. 2016. JSpeciesWS: a web server for prokaryotic species circumscription based on pairwise genome comparison. Bioinformatics 32:929-31.
7. Sullivan MJ, Petty NK, Beatson SA. 2011. Easyfig: a genome comparison visualizer. Bioinformatics 27:1009-10.
8. Lorenz R, Bernhart SH, Höner zu Siederdissen C, Tafer H, Flamm C, Stadler PF, Hofacker IL. 2011. ViennaRNA Package 2.0. Algorithms for molecular biology 6:1-14.
9. Ontiveros-Palacios N, Cooke E, Nawrocki EP, Triebel S, Marz M, Rivas E, Griffiths-Jones S, Petrov AI, Bateman A, Sweeney B. 2025. Rfam 15: RNA families database in 2025. Nucleic Acids Res 53:D258-D267.
10. Wu T, Hu E, Xu S, Chen M, Guo P, Dai Z, Feng T, Zhou L, Tang W, Zhan L, Fu X, Liu S, Bo X, Yu G. 2021. clusterProfiler 4.0: A universal enrichment tool for interpreting omics data. Innovation (Camb) 2:100141.
11. Gilchrist CL, Chooi Y-H. 2021. Clinker & clustermap. js: automatic generation of gene cluster comparison figures. Bioinformatics 37:2473-2475.
12. Moreira SM, Chyou TY, Wade JT, Brown CM. 2024. Diversification of the Rho transcription termination factor in bacteria. Nucleic Acids Res 52:8979-8997.
13. Jeong Y, Hong SJ, Cho SH, Yoon S, Lee H, Choi HK, Kim DM, Lee CG, Cho S, Cho BK. 2021. Multi-Omic Analyses Reveal Habitat Adaptation of Marine Cyanobacterium Synechocystis sp. PCC 7338. Front Microbiol 12:667450.
14. Rosinski-Chupin I, Sauvage E, Sismeiro O, Villain A, Da Cunha V, Caliot ME, Dillies MA, Trieu-Cuot P, Bouloc P, Lartigue MF, Glaser P. 2015. Single nucleotide resolution RNA-seq uncovers new regulatory mechanisms in the opportunistic pathogen Streptococcus agalactiae. BMC Genomics 16:419.
15. Rosinski-Chupin I, Sauvage E, Fouet A, Poyart C, Glaser P. 2019. Conserved and specific features of Streptococcus pyogenes and Streptococcus agalactiae transcriptional landscapes. BMC Genomics 20:236.
16. You L, Omollo EO, Yu C, Mooney RA, Shi J, Shen L, Wu X, Wen A, He D, Zeng Y, Feng Y, Landick R, Zhang Y. 2023. Structural basis for intrinsic transcription termination. Nature 613:783-789.

17. Dar D, Sorek R. 2018. High-resolution RNA 3'-ends mapping of bacterial Rho-dependent transcripts. Nucleic Acids Res 46:6797-6805.
18. Kobayashi H, Akitomi J, Fujii N, Kobayashi K, Altaf-Ul-Amin M, Kurokawa K, Ogasawara N, Kanaya S. 2007. The entire organization of transcription units on the Bacillus subtilis genome. BMC Genomics 8:197.
19. Ju X, Li D, Liu S. 2019. Full-length RNA profiling reveals pervasive bidirectional transcription terminators in bacteria. Nature microbiology 4:1907-1918.
20. Kim S-H, Mamuad LL, Islam M, Lee S-S. 2020. Reductive acetogens isolated from ruminants and their effect on in vitro methane mitigation and milk performance in Holstein cows. Journal of Animal Science and Technology 62:1.
21. Kolmogorov M, Yuan J, Lin Y, Pevzner PA. 2019. Assembly of long, error-prone reads using repeat graphs. Nat Biotechnol 37:540-546.
22. Hunt M, Silva ND, Otto TD, Parkhill J, Keane JA, Harris SR. 2015. Circlator: automated circularization of genome assemblies using long sequencing reads. Genome Biol 16:294.
23. Tatusova T, DiCuccio M, Badretdin A, Chetvernin V, Nawrocki EP, Zaslavsky L, Lomsadze A, Pruitt KD, Borodovsky M, Ostell J. 2016. NCBI prokaryotic genome annotation pipeline. Nucleic Acids Res 44:6614-24.
24. Choe D, Szubin R, Poudel S, Sastry A, Song Y, Lee Y, Cho S, Palsson B, Cho BK. 2021. RiboRid: A low cost, advanced, and ultra-efficient method to remove ribosomal RNA for bacterial transcriptomics. PLoS Genet 17:e1009821.
25. Dar D, Shamir M, Mellin JR, Koutero M, Stern-Ginossar N, Cossart P, Sorek R. 2016. Term-seq reveals abundant ribo-regulation of antibiotics resistance in bacteria. Science 352:aad9822.
26. Anders S, Huber W. 2010. Differential expression analysis for sequence count data. Nature Precedings doi:10.1038/npre.2010.4282.1.
27. Kanehisa M, Sato Y, Morishima K. 2016. BlastKOALA and GhostKOALA: KEGG Tools for Functional Characterization of Genome and Metagenome Sequences. J Mol Biol 428:726-731.
28. Jeong Y, Kim JN, Kim MW, Bucca G, Cho S, Yoon YJ, Kim BG, Roe JH, Kim SC, Smith CP, Cho BK. 2016. The dynamic transcriptional and translational landscape of the model antibiotic producer Streptomyces coelicolor A3(2). Nat Commun 7:11605.
29. Lee Y, Lee N, Jeong Y, Hwang S, Kim W, Cho S, Palsson BO, Cho B-K. 2019. The transcription unit architecture of Streptomyces lividans TK24. Frontiers in Microbiology 10:2074.
30. Hwang S, Lee N, Choe D, Lee Y, Kim W, Jeong Y, Cho S, Palsson BO, Cho B-K. 2021. Elucidating the regulatory elements for transcription termination and posttranscriptional processing in the Streptomyces clavuligerus genome. Msystems 6:10.1128/msystems. 01013-20.
31. Bailey TL, Boden M, Buske FA, Frith M, Grant CE, Clementi L, Ren J, Li WW, Noble WS. 2009. MEME SUITE: tools for motif discovery and searching. Nucleic Acids Res 37:W202-8.
32. Crooks GE, Hon G, Chandonia JM, Brenner SE. 2004. WebLogo: a sequence logo generator. Genome Res 14:1188-90.
33. Grant CE, Bailey TL, Noble WS. 2011. FIMO: scanning for occurrences of a given motif. Bioinformatics 27:1017-8.

34. Long CP, Antoniewicz MR. 2019. High-resolution (13)C metabolic flux analysis. Nat Protoc 14:2856-2877.
